# Computer-aided engineering of a branching sucrase for the glucodiversification of a tetrasaccharide precursor of *S. flexneri* antigenic oligosaccharides

**DOI:** 10.1038/s41598-021-99384-9

**Published:** 2021-10-13

**Authors:** Mounir Benkoulouche, Akli Ben Imeddourene, Louis-Antoine Barel, Dorian Lefebvre, Mathieu Fanuel, Hélène Rogniaux, David Ropartz, Sophie Barbe, David Guieysse, Laurence A. Mulard, Magali Remaud-Siméon, Claire Moulis, Isabelle André

**Affiliations:** 1 Toulouse Biotechnology Institute, TBI, Université de Toulouse, CNRS, INRAE, INSA, 135, Avenue de Rangueil, 31077 Toulouse Cedex 04, France; 2 Institut Pasteur, CNRS UMR3523 Unité de Chimie des Biomolécules, 28 Rue du Dr Roux, 75724 Paris Cedex 15, France; 3 Université Paris Descartes, Sorbonne Paris Cité, Paris, France; 4 INRAE, UR BIA, 44316 Nantes, France; 5 INRAE, BIBS Facility, 44316 Nantes, France

**Keywords:** Biocatalysis, Carbohydrates, Enzymes, Biochemistry, Biotechnology, Evolution, Molecular biology

## Abstract

Enzyme engineering approaches have allowed to extend the collection of enzymatic tools available for synthetic purposes. However, controlling the regioselectivity of the reaction remains challenging, in particular when dealing with carbohydrates bearing numerous reactive hydroxyl groups as substrates. Here, we used a computer-aided design framework to engineer the active site of a sucrose-active $\mathrm{\alpha }$-transglucosylase for the 1,2-*cis*-glucosylation of a lightly protected chemically synthesized tetrasaccharide, a common precursor for the synthesis of serotype-specific *S. flexneri* O-antigen fragments. By targeting 27 amino acid positions of the acceptor binding subsites of a GH70 branching sucrase, we used a RosettaDesign-based approach to propose 49 mutants containing up to 15 mutations scattered over the active site. Upon experimental evaluation, these mutants were found to produce up to six distinct pentasaccharides, whereas only two were synthesized by the parental enzyme. Interestingly, we showed that by introducing specific mutations in the active site of a same enzyme scaffold, it is possible to control the regiospecificity of the 1,2-*cis* glucosylation of the tetrasaccharide acceptor and produce a unique diversity of pentasaccharide bricks. This work offers novel opportunities for the development of highly convergent chemo-enzymatic routes toward *S. flexneri* haptens.

## Introduction

In recent years, enzyme engineering has enabled unprecedented expansion of the enzyme repertoire, notably endowed with desirable physical and catalytic properties, and broadened the horizons of enzyme-based process development^1–3^. In particular, progress in the development of bioinformatics tools and computational methods has considerably contributed to better understand natural evolution of enzymes, rationalize mutations to acquire a given function or property and *in fine* accelerate conception of tailored catalysts for synthetic purposes. The strength of computational protein design has been demonstrated to engineer enzymes with improved stability^4,5^, enhanced catalytic performances^6^, altered substrate selectivity and specificity^7^, or even able to catalyze new-to-Nature reactions^8–11^. Importantly, these computer-aided engineering methods enable to fine-tune the active site toward recognition and conversion of exogenous substrates by few specific amino acid mutations^9,12–15^, thereby avoiding the extensive screening of large libraries required in directed evolution approaches. This is particularly advantageous when the amount of available substrate is limited, especially when rare molecules that are difficult to obtain or synthesize are targeted, or when no high-throughput assay system is available. For many years, our research group has applied such rational and semi-rational engineering approaches to engineer sucrose-active α-transglucosylases from Glycoside Hydrolase (GH) families 13 and 70 of the CAZy classification^16^ in order to produce a variety of glycoconjugates, carbohydrate derivatives, including chemically modified oligosaccharide precursors^15,17–19^. In the context of carbohydrate synthesis, the structural complexity renders particularly challenging differentiation and characterization of products obtained from catalytic reaction. Therefore, low throughput screening methods based on chromatography, mass spectrometry, and NMR spectroscopy are usually used.

**Figure 1:**
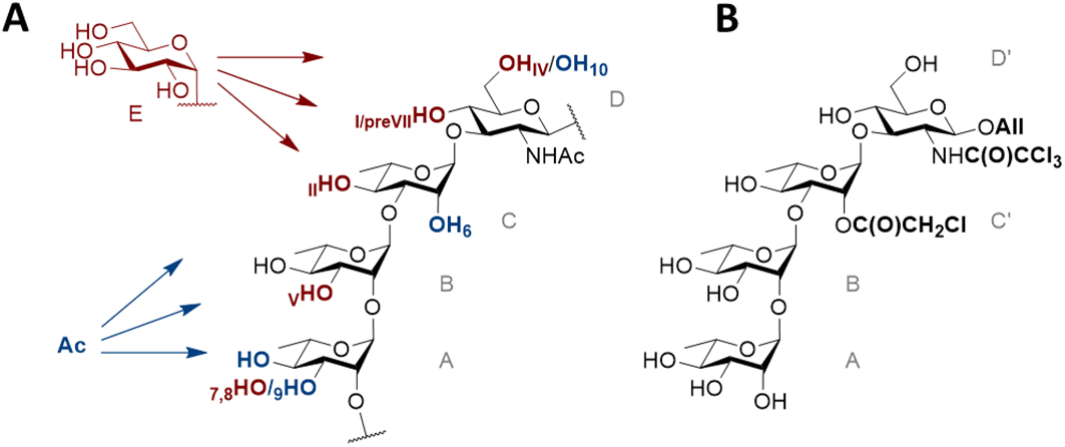
(**A**) Overview of the repeating units of most *S. flexneri* O-Ags showing the tetrasaccharide backbone **ABCD** and sites of $\mathrm{\alpha }$-d-glucosylation (**E**, in red) and O-acetylation (Ac, in blue) ^22^. Each glucosylation pattern is associated to the relevant *S. flexneri* type-specific (Roman numeral) and group-specific (Arabic numeral) antigenic determinants. (**B**) The lightly protected tetrasaccharide **ABC’D’** mimicking **ABCD** and used in this study as a glucansucrase acceptor substrate to access *S. flexneri* representative oligosaccharides by a chemoenzymatic route. The chloroacetyl group at OH-2_C_ paves the way to a hydroxyl group or an acetate (group O-factor 6) as required. The allyl aglycon and 2_D_-trichloroacetamide were introduced at an early stage in view of future chain elongation. All free hydroxyl groups are potential sites of enzymatic glucosylation with modifications at 3_A_, 3_B_, 4_C_, 4_D_ and 6_D_ featuring *S. flexneri* type/group-specific branching points. All: allyl.

In the continuation of our efforts^15,18,20,21^ to fashion the main components of the *Shigella flexneri* lipopolysaccharide^22,23^, the work disclosed herein aims at developing novel chemo-enzymatic routes to access well-defined O-antigen (O-Ag) fragments that could enter in the composition of broad serotype coverage vaccines^24^. *Shigella* are gram negative bacteria responsible for shigellosis, a diarrheal disease that represents a major burden in low and middle income countries^25^, and for which there is no vaccine yet. The *S. flexneri* O-Ag is the primary target of protection acquired upon natural infection and has attracted major interest in vaccine design^26^. Polysaccharide conjugate vaccine candidates issued from a variety of technologies are being developed^27^. In particular, we have previously reported a synthetic carbohydrate-based conjugate vaccine targeting *S. flexneri* 2a, the most prevalent *S. flexneri* serotype. This vaccine candidate^28^ was safe and well tolerated and demonstrated promising immunogenicity data in Human^29^. Those achievements support further investigation on the potential of the strategy to ensure an acceptable serotype coverage. Among the prevalent isolates observed in *Shigella* infection are found *S. flexneri* serotypes which present a tremendous O-Ag structural diversity. While most *S. flexneri* O-Ags share the same linear backbone composed of 1,2-*trans* linked l-rhamnose (**A**, **B**, **C**) and *N*-acetyl-d-glucosamine (**D**) residues, the repeating units of the different serotype-specific O-Ags differ for the most part by their α-d-glucosylation (**E**) pattern and by their *O*-acetyl substitutions whether stoichiometric or not (Fig. 1).

One main synthetic roadblock to access chemically defined *S. flexneri* serotype-specific oligosaccharides resides in the regio- and stereo-specific control of the 1,2-*cis* glucosylation step. With the aim of proposing novel and easy-to-implement synthetic strategies, we came up with the idea of developing a highly convergent chemo-enzymatic strategy that will target the enzymatic regioselective glucosylation of a lightly protected **ABC’D’** tetrasaccharide (allyl α-l-rhamnopyranosyl-(1 → 2)-α-l-rhamnopyranosyl-(1 → 3)-2-*O*-chloroacetyl-α-l-rhamnopyranosyl-(1 → 3)-2-deoxy-2-trichloroacetamido-β-d-glucopyranoside) resembling the common backbone repeating unit of most *S. flexneri* O-Ags^24^ (Fig. 1). Using native branching sucrases from the GH70 family, we previously reported the successful enzymatic glucosylation of **ABC’D’** for the production of a pentasaccharide **P1** identified as **ABC’[E(1 → 6)]D’**, characteristic of serotypes 4a/4b^24^. Among the six tested branching sucrases, the best conversion of **ABC’D’** (31%) was obtained with BRS-B Δ1 enzyme. Using a pre-existing library of mutants of another branching sucrase, namely ΔN_123_-GBD-CD2, we later obtained three additional pentasaccharides (named **P2**, **P2’** and **P3**), of which **P2** and **P2’** were fully characterized as [**E(1 → 3)]ABC’D’** and [**E(1 → 4)]ABC’D’**, respectively^21^. Of interest, **P2** was found to be representative of *S. flexneri* serotype 3a, one of the most prevalent *Shigella* serotypes.

In order to further extend the structural diversity of glucansucrase-mediated accessible pentasaccharides, we undertook the challenge of re-designing the active site of a branching sucrase derived from BRS-B, found so far to be the most efficient enzyme for the glucosylation of **ABC’D’**. The construction of a 3D model by comparative modelling enabled the use of structure-based engineering strategies. Using a combination of molecular modelling methods and the RosettaDesign-based approach^30^, we explored amino acid mutations in the active site that could favor catalytically productive binding of **ABC’D’** in various orientations to enable the regioselective $\mathrm{\alpha }$-d-glucosylation of residues **A**, **C’** or **D’** of the non-natural lightly protected tetrasaccharide. Enzymatic glucosylation products characteristic of prevalent *S. flexneri* serotypes: **ABC’[E(1 → 4)]D’** (*S. flexneri* 1a/1b, type I), **AB[E(1 → 4)]C’D’** (*S. flexneri* 2a/2b, type II) and **[E(1 → 3)]ABC’D’** (*S. flexneri* 3a/2b, group O-factor 7,8) were targeted in priority. Evidently, conservation of the original specificity for the sucrose donor substrate was also a main concern during the redesign of the enzyme active site. Extensive redesign led to the selection of 49 mutants containing up to 15 mutations scattered over 27 amino acid positions of the acceptor binding subsites. Mutants were constructed and assayed for glucosylation of **ABC’D’**. Our results show the versatility of the BRS-B scaffold, which by the introduction of specific combinations of mutations in the active site gave access to a broad range of 1,2-*cis* glucosylation patterns from a common acceptor molecule.

## Results and discussion

### Computer-aided re-design of BRS-B Δ2 branching sucrase active site

**Figure 2:**
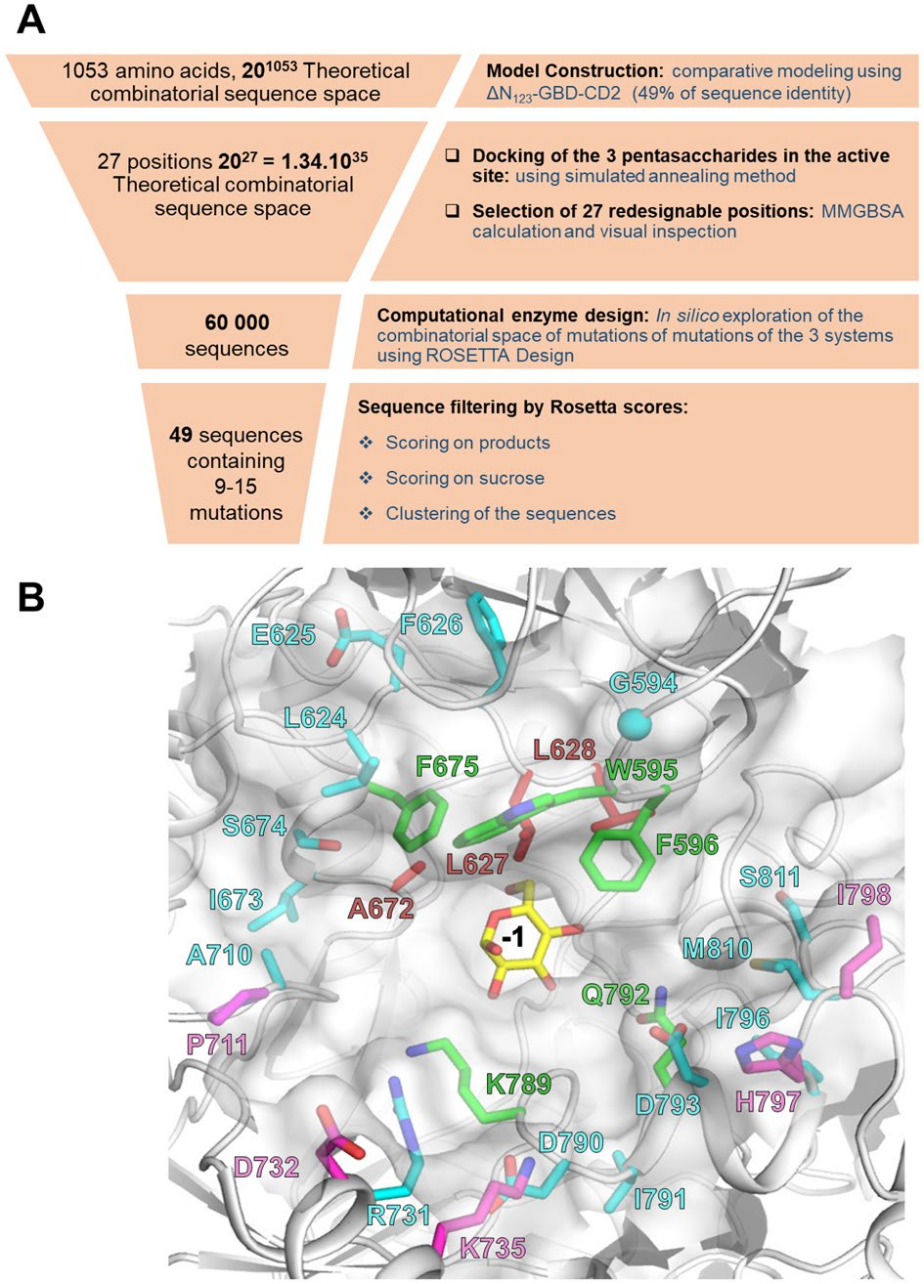
(**A**) Computer-aided approach for BRS-B Δ2 active site re-design. After successive steps, the final designed library contained 49 sequences featuring between 9 and 15 mutations in the active site, each. (**B**) View of the active site of BRS-B Δ2 model. The 27 redesignable amino acids are shown in red, green, cyan and magenta according to their belonging to the first, second, third and fourth contact shell, respectively, surrounding the glucosyl residue (yellow sticks) in the -1 position as extracted from the structure of inactive GTF-180 in complex with sucrose (PDB ID: 3HZ3). The docked pentasaccharides are not shown for clarity purpose. Molecular graphics were prepared using PYMOL 1.7 (PyMOL Molecular Graphics System, Schrödinger, LLC).

**Figure 3:**
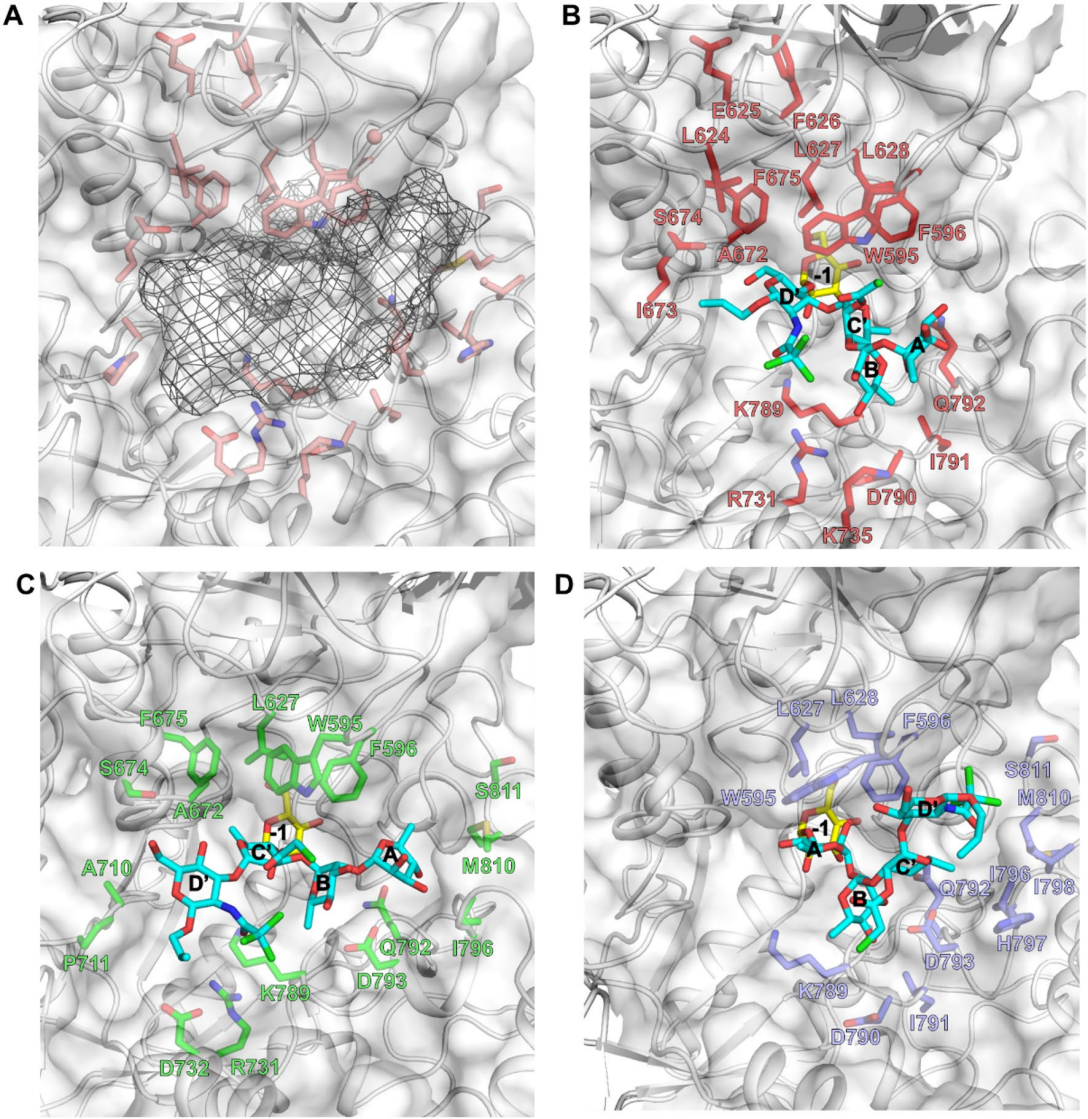
View of the space occupied by the three gathered pentasaccharides targeted by the design bound in the active site of parental BRS-B Δ2 (**A**) as well as the targeted amino acids in each of the designs: **ABC’[E(1 → 4)]D’** (*S. flexneri* 1a/1b) targeted by mutants M1-M16 from Group I is shown in (**B**); **AB[E(1 → 4)]C’D’** (*S. flexneri* 2a) targeted by mutants M17-M34 from Group II is shown in (**C**); **[E(1 → 3)]ABC’D’** (*S. flexneri* 3a) targeted by mutants M35-M49 from Group III is shown in (**D**). Molecular graphics were prepared using PYMOL 1.7 (PyMOL Molecular Graphics System, Schrödinger, LLC).

With the aim of performing site-selective $\mathrm{\alpha }$-d-glucosylation of tetrasaccharide **ABC’D’** and thus enlarging accessible *S. flexneri* pentasaccharide diversity, we focused our work on the redesign of the active site of a branching sucrase from *Leuconostoc citreum* NRRL B-742 named BRS-B. To facilitate recombinant enzyme expression in *Escherichia coli*, a truncated variant -called BRS-B Δ2- was constructed by removing 153 amino acids from the N-terminus of BRS-B Δ1 enzyme^31^. This variant showed the same specific activity as the parental BRS-B enzyme or BRS-B Δ1^24^ and had better soluble expression (data not shown). As the three-dimensional structure of this enzyme was unknown, we decided to construct a 3D-model of BRS-B Δ2 using as template the branching sucrase ΔN_123_-GBD-CD2, for which an X-ray structure is available (PDB ID: 3TTQ). BRS-B Δ2 contains 1053 amino acid residues and shares 49% identity with ΔN_123_-GBD-CD2. When considering only the active site of the enzymes, the sequence identity increases to 60%, indicating a high conservation of active site residues. This allowed construction of a 3D-model of BRS-B Δ2 by comparative modelling and opened the way to computer-aided design approaches illustrated in the framework of Fig. 2A. Using the 3D model, pentasaccharide products characteristic of prevalent *S. flexneri* serotypes, **ABC’[E(1 → 4)]D’** (*S. flexneri* 1a/1b), **AB[E(1 → 4)]C’D’** (*S. flexneri* 2a) and **[E(1 → 3)]ABC’D’** (*S. flexneri* 3a), were docked in the active site (Fig. 3). The crystallographic structure of homologous GTF180 glucansucrase (PDB ID: 3HZ3) in complex with sucrose bound in the active site^32^ was used as template to guide docking of the different pentasaccharides. More particularly, pentasaccharides were initially constructed using 3D coordinates of the sucrose glucosyl moiety from the crystallographic complex. Systems were subsequently subjected to simulated annealing (from 0 to 350 K in 100 ps and vice versa) in vacuum with harmonic positional restraints of 50.0 kcal/mol/Å^2^ on the enzyme, the glucosyl unit, and the sugar pucker rings of **ABC’D’**. The lowest energy systems from each simulated annealing were then selected as starting points for the computational enzyme design procedure. After excluding the catalytic residues (the nucleophilic D671, the acid/base E709 and the transition state stabilizer D1136) and other amino acid residues described as important for either catalysis or sucrose recognition (R669, H787, Y144 and D1183 identified by homology with GTF-180)^32^, we selected 27 mutable (or designable) positions in total: 24 in catalytic domain A and 3 in domain B—(G594, W595 and F596 residues) (Fig. 2B) on the basis of Molecular Mechanics/Generalized Born Surface Area (MM/GBSA) calculation (Figure S1) and careful visual inspection.

Most of these positions belong to loops with the exception of R731, D732, K735, D793 and H797, which are located in α-helices. Overall, these 27 residues are scattered over the catalytic site along four successive contact shells, due to the sliding of the **ABC’D’** core with respect to the -1 subsite where the glucosyl residue is bound (Fig. 2B). Moreover, all selected residues are found highly variable among all known GH70 enzymes with the exception of amino acids L627 and A672 from the first shell which are strictly conserved (Table S1). It should be noticed that, if all 27 positions were considered to be mutable by one of the 20 possible amino acids, the theoretical combinatorial sequence space would have been as large as 1.34 × 10^35^, clearly out of reach of currently available experimental screening methods and computational approaches. To explore and reduce this combinatorial space, we thus considered an enzyme design protocol based on the sampling of mutations at each of the 27 positions by performing 60,000 independent runs (20,000 per system) of Rosetta *Enzyme_Design* (https://www.rosettacommons.org/), which considers the backbone and side chain flexibility of amino acids. The designed sequences were subsequently filtered using the Rosetta scores corresponding to enzyme:pentasaccharide binding interaction for each of the three systems, followed by a second round of sequence filtering based on the docking of sucrose donor in each mutant and estimation of its binding interaction (Figure S2). Finally, in order to limit sequence redundancy and enhance sequence variability in the final set of sequences, all designed sequences were clustered based on the percentage of sequence identity. The best Rosetta scores (with respect to pentasaccharides and sucrose) of each cluster (total of 49) were then selected for experimental evaluation. The set of the corresponding 49 sequences (Table S2) contained between 9 and 15 mutations located between positions 595 and 811. These sequences were classified in three groups depending on the targeted pentasaccharide: group I (M1 to M16) for **ABC’[E(1 → 4)]D’** (*S. flexneri* 1a/1b), group II (M17 to M34) for **AB[E(1 → 4)]C’D’** (*S. flexneri* 2a) and group III (M35 to M49) for **[E(1 → 3)]ABC’D’** (*S. flexneri* 3a) (Fig. 3).

### Recombinant production of mutants

When using conditions for recombinant expression in *E. coli* of parental BRS-B Δ2 enzyme*,* formation of inclusion bodies was observed for the 49 mutants. In order to enhance solubilization and prevent aggregation, we selected at random the clones expressing mutants M14 and M34, which contained 15 and 11 mutations, respectively, and attempted their production in the presence of different combinations of chaperone proteins^33^ (Table S3), as well as by growing the cells at 21 °C and using another optimized auto-inducible medium^34^. Using co-expression with plasmids pG-KJE8 (coding for dnaK, dnaJ, grpE, groES, groEL chaperones) or pTf16 (coding for tig), mutant proteins were partially recovered in the soluble fraction, showing the highest amount of soluble proteins after 24 h of culture when using plasmid pTf16, and 32 h when using plasmid pG-KJE8. The optimization of the soluble expression of mutant M21 is presented in Figure S3 as an example. Based on these results, the 49 mutants were successfully produced using the optimized culture conditions and chaperones encoded by either plasmid pTf16 or pG-KJE8.

### Screening of the mutant library for the ability to use sucrose as donor substrate

**Figure 4:**
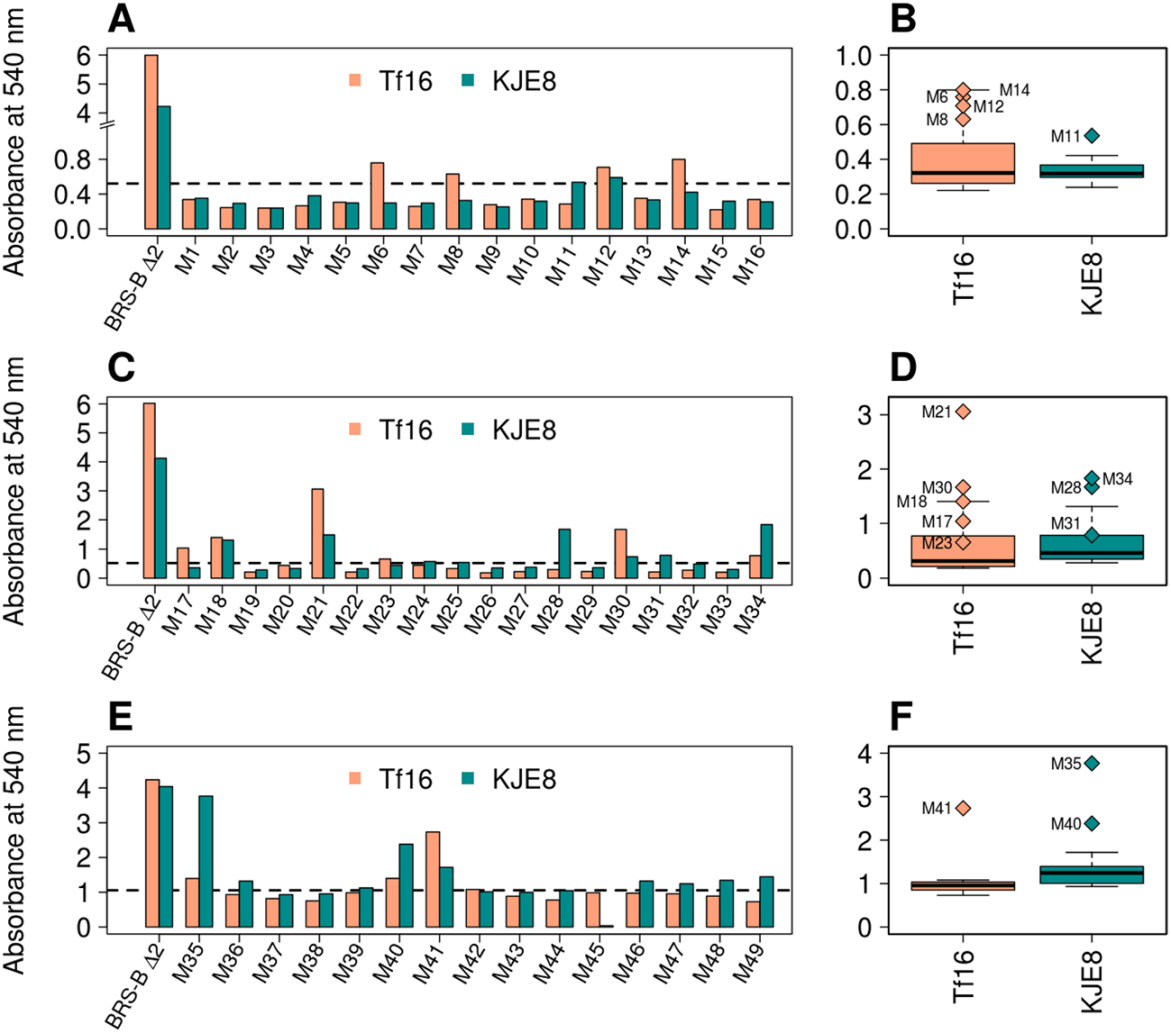
(**A**, **C**, **E**): Absorbance at 540 nm determined after the reducing sugar “ON/OFF” assay using crude enzyme extracts of the 49 mutants co-produced with either *tig* (Tf16, light salmon) or *dnaK*, *dnaJ*, *grpE*, *groES*, *groEL* chaperone proteins (KJE8, dark cyan) in presence of 100 g L^−1^ sucrose during 70 h incubation. The parental enzyme BRS-B Δ2 is indicated as a reference. Dashed lines indicate absorbance level of the inactive mutant BRS-B Δ2 E709Q. (**B**, **D**, **F**) boxplot representing the absorbance data. Parental enzyme BRS-B Δ2 was excluded from the analysis. Data points (diamonds) pinpoint the 16 mutants selected for further evaluation.

In order to assess the capacity of the mutants to utilize sucrose, a colorimetric “ON/OFF” assay was set up on sucrose as sole substrate, based on the use of dinitrosalicylic acid (DNS) to measure the amount of reducing sugars (i.e. fructose, glucose) released after substrate hydrolysis. The soluble fractions were incubated for 70 h with substrate and reducing sugar production was determined at 540 nm. As shown Fig. 4-panels A, C, and E, all the mutants were strongly impacted for sucrose consumption compared to the parental enzyme.

Out of the 49 mutants, the boxplot analysis enabled us to retain 16 mutants found in the top tertile (and above) of the absorbance boxplot (Fig. 4 panels B, D and F). These 16 mutants were purified to homogeneity. Among them, four mutants quickly aggregated after purification (M8, M12 and M17 produced with Tf16 and M11 produced with KJE8), indicating that their stability was impacted by the mutations. Active mutants (12 out of 49 screened, 24.5% active mutants in total) belonged to all three groups, with 2 from group I, 7 from group II, and 3 from group III.

These 12 mutants (M6, M14, M18, M21, M23, M30 and M41 produced with Tf16 and M28, M31, M34, M35 and M40 produced with KJE8) were retained for further evaluation of their ability to glucosylate **ABC’D’**. Meanwhile, the specific activity toward sucrose was found to be only 1.9% of that of the parental enzyme for M21, 0.2% for M23 1.2% for M30, 0.1% for M34, and 1% for M35 (Table 1). The specific activity of the other mutants (M6, M14, M18, M28, M31 and M40) could not be determined, indicating a tremendous loss of activity toward sucrose donor substrate for all these mutants.

**Table 1:** Specific activity on sucrose (in U mg^−1^ of purified enzyme) was determined for mutants M21, M23, M30, M34 and M35. ^a^ data from ref ^35^. *n.d.*: not determined.

Enzyme	BRS-B Δ2	M21	M23	M30	M34	M35
Specific activity on sucrose (U.mg^−1^ of purified enzyme)	26^a^	0.46	0.06	0.30	0.02	0.24

### Transglucosylation of tetrasaccharide ABC’D’

**Figure 5:**
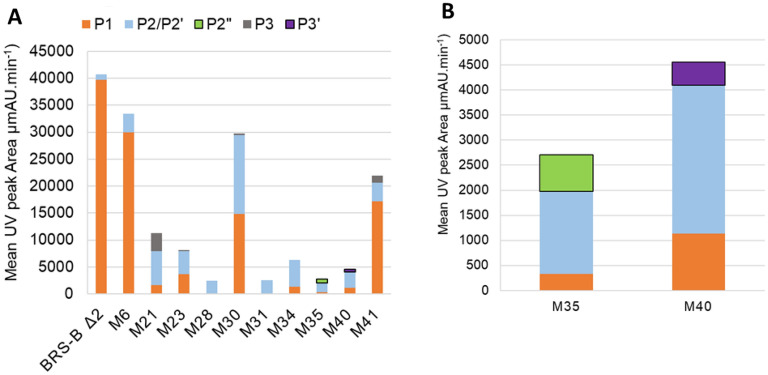
(**A**) Distribution of the products formed by BRS-B Δ2 enzyme and its mutants, and determined from HPLC–UV analyses of 16 h reaction mixture with 50 mM **ABC’D’**, 1 M sucrose at pH 5.75 and variable amounts of purified enzymes in 50 µL reaction volume. Mass Spectrometry revealed a mass increase of 162 g mol^−1^ compared to **ABC’D’**, corresponding to mono-glucosylated tetrasaccharides. M14 and M18 did not produce any pentasaccharide product (not shown). (**B**) An enlarged view of the products (notably novel products **P2″** and **P3’**) formed by M35 and M40 is shown on the right panel.

All 12 selected mutants were tested for their ability to glucosylate tetrasaccharide **ABC’D’** and their products were analyzed by LC–MS. Reaction pH was set to 5.75 despite the **ABC’D’** stability decrease at this pH^24^, due to the quick aggregation of mutants when lowering the pH. With the exception of two mutants (M14 and M18), all selected mutants were able to transfer glucosyl moieties onto **ABC’D’**, yielding at least one and up to four mono-glucosylated products (Fig. 5). Overall, six different pentasaccharide products were detected, which were named **P1**, **P2**, **P2’**, **P2’’**, **P3** and **P3’** based on their RP-HPLC retention time (*t*_*r*_) and molecular mass (Figure S4). **P2** and **P2’** are co-eluted products (*t*_*R*_ = 21.9 min) hardly distinguishable by LC–MS. However their chemical structures have been characterized by NMR spectroscopy as being distinct pentasaccharides in prior work^24^. Mutant M6 showed a profile similar to that of the parental enzyme BRS-B Δ2 and produced mainly **P1** (*t*_*R*_ = 21.6 min) together with a small amount of **P2/P2’**. Relatively, mutant M34 produced significantly lower amounts of **P1** and increased amounts of **P2/P2’**. M28 and M31 were found to produce only **P2/P2’**.

Another group of mutants composed of M21, M23, M30 and M41 shared a common product profile (Fig. 5). While they all produced various ratio of products **P1** and **P2/P2’**, they also formed a novel pentasaccharide named **P3**, seemingly not at all synthesized by the parental enzyme. Mutant M21 was found to produce **P3** in larger amount. Interestingly, based on its retention time (*t*_*R*_ = 16.3 min), **P3** was found to be also produced by mutant W2135S-F2136L of the enzyme ΔN_123_-GBD-CD2 in our earlier report^21^. In addition, two compounds with distinct retention times compared to already characterized pentasaccharides are observed leading us to assume the formation of novel compounds which were named **P2″** (*t*_*R*_ = 22.1 min) and **P3’** (*t*_*R*_ = 15.3 min). While **P2″** is observed for M35, **P3’** is produced in trace amounts by M40.

### Structural characterization of the formed products

Mutant M21 forming products **P1**, **P2**/**P2’** and **P3** was selected to produce sufficient amounts of pentasaccharides for structural characterization using high field NMR spectroscopy. M21 was produced and purified to homogeneity and used to carry out a 1 mL-scale reaction in presence of **ABC’D’** and sucrose. The structure of **P1** was previously determined without ambiguity^24^, therefore only products **P2**/**P2’** (5.0 mg) and **P3** (1.6 mg) were isolated. The 1D ^1^H NMR spectrum obtained for **P2**/**P2’** isolated using M21 was perfectly superimposed to the one previously obtained using mutant F2163G of ΔN_123_-GBD-CD2^21^ (Figure S5). This confirmed that glucosylation occurred on OH-4_A,_ corresponding thus to **P2’**_._ The analysis of the HSQC spectra of **P3** showed shifted resonances of the glucosylated positions and adjacent atoms (Figure S6); the glucosylated 4_B_ carbon was high frequency shifted from 72.2 ppm to 80.9 ppm, while the adjacent 3_B_ and 5_B_ were shifted to lower frequency, from 69.8 ppm and 69.6 ppm to 68.6 ppm and 68.4 ppm, respectively. The cross-correlation peaks of the shifted **B** and **E** carbon resonances were assigned using Double Quantum Filtered COrrelation (QDF COSY) (Figure S7). **P3** was thus confirmed as being glucosylated on OH-4_B_.

Given the very low amounts of **P2″** and **P3’** pentasaccharides produced by mutant M35 and M40, respectively, we turned to a mass spectrometry (MS) based approach for their identification. This analytical method is more sensitive (requires a few tens of ng of compounds), avoids laborious production and purification steps by enabling the analyses of complex mixtures coupled with appropriate UHPLC method, and allows a structural characterization by using tandem MS (MS/MS). For reference purpose, the M21 reaction mixture was analyzed in the same conditions. UHPLC-MS analysis of M21, M35 and M40 reaction mixtures using a porous graphitized carbon column (Figure S8) revealed the presence of three pentasaccharide isomers produced by M21 (*t*_*R*_ = 21.12 min, *t*_*R*_ = 22.70 min, *t*_*R*_ = 22.93 min) and four isomers independently produced by M35 and M40 (*t*_*R*_ = 21.16 min, *t*_*R*_ = 21.96 min, *t*_*R*_ = 22.07 min and *t*_*R*_ = 22.71 min for M35 ; *t*_*R*_ = 21.16 min, *t*_*R*_ = 21.90 min, *t*_*R*_ = 22.71 min and *t*_*R*_ = 22.86 min for M40). Each of these species was characterized by UHPLC-MS/MS using collision induced dissociation (CID). By this approach, we faced the high lability of the chloroacetyl moiety. The collision energy had to be adjusted compared to classical MS/MS CID based approach to allow the production of interesting fragments. This explained that we had to magnify the low mass area on the figures below *m/z* 825, blue part) on the spectra for each species.

First, the structure of the produced molecules shared between M35 and M40 was studied in parallel with the HPLC–UV, NMR and UHPLC-MS data. Indeed, tandem mass spectrometry data using CID fragmentation is not sufficient on its own to characterize all the structural details. For example, tandem MS spectrum of the product **P1** (M35-4 and M40-3, glucosylated on OH-6_D’_) at *t*_*R*_ = 22.71 min is shown Figure S9. If it remains impossible to discriminate the branching of the hexose between the 4 or 6 hydroxyl groups of the glucosamine **D′**, the parallel with the NMR data confirmed the glucosylation on OH-6_D’_. The MS/MS spectrum of the product at *t*_*R*_ = 21.96 for M35-2 and *t*_*R*_ = 21.90 for M40-2 (the slight shift in retention time between the two samples for **P2/P2’** can be explained by the presence of the partially co-eluted species is presented Figure S10). As shown by the HPLC–UV analysis, the OH-4_A_ is absent in the samples so **P2** was confirmed as being glucosylated on OH-3_A_. The MS/MS analysis of product M35-1 and M40-1 at *t*_*R*_ = 21.16 min (Figure S8) is presented Figure S11. This analysis confirms a glucosylation on rhamnose **B** but it was impossible to decipher between glucosylation on OH-3_B_ (**P3’**) or OH-4_B_ (**P3**) from the CID experiments. However, from the HPLC–UV, NMR and UHPLC-MS data of the M21 reaction mixture, with M21-1 eluting at *t*_*R*_ = 22.12 min, we assigned the **P3** product at *t*_*R*_ = 21.16 min (Figure S8). Interestingly, **P3** was not detected in the M35 samples using HPLC–UV analysis. This reveals that the UHPLC-MS approach is more sensitive and can reveal a highest diversity in biological medium.

**Figure 6:**
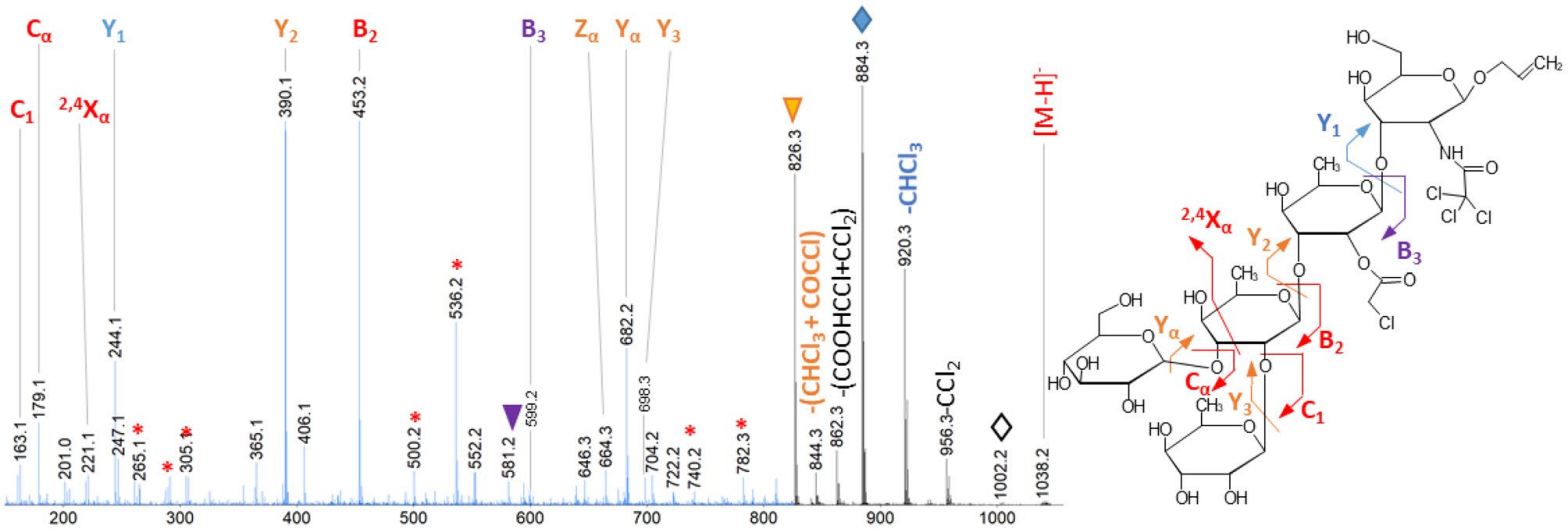
UHPLC-ESI–MS/MS spectrum of the pentasacharide M40-4 isolated as [M-H]^−^ at *m/z* 1038.21 at *t*_*R*_ = 22.86 min in samples from M40 validated as being substituted at OH-3_B_ (**P3’**). The blue area of the spectrum is enlarged by a factor of 4 in the intensity axis. Annotations in red correspond to intact product ions. Annotation in blue correspond to product ions with a loss of CHCl_3_. Annotations in purple correspond to product ions with a loss of COCHCl. Annotation in orange correspond to product ions with a loss of CHCl_3_ + COHCl_3_. All these labile function losses are in agreement with the structure. Red * indicate consecutive fragmentations, ▽ indicate H_2_O loss, ◊ indicate HCl loss. For clarity, only one fragment per pair (B, C and Y, Z) was reported on the annotated structure.

Concerning the molecules specific of each sample, the MS/MS spectrum analysis of the last eluted molecule M40-4 at *t*_*R*_ = 22.86 min is shown in Fig. 6. As discussed previously, CID experiments did not allow to differentiate glucosylation on OH-3_B_ (**P3’**) or OH-4_B_ (**P3**). However, by deduction from the identification of **P3** at *t*_*R*_ = 21.16 min, the positioning of the branched hexose on rhamnose **B** of the tetrasaccharide was validated as being OH-3_B_. This new product corresponds to **P3'**.

The MS/MS spectrum analysis of the partially coeluted species M35-3 at *t*_*R*_ = 22.07 min is shown Fig. 7A. We integrated only the end of the chromatographic peak in order to exclude a cross contamination with the fragments acquired for the **P2** species at *t*_*R*_ = 21.96 min. The fragmentation spectrum obtained is really close from the MS/MS data obtained for the oligosaccharide **P2** (glucosylation on OH-3_A_, Figure S10). However, as illustrated in Fig. 7B and Fig. 7C, there is a significant difference in the low mass range between the spectra acquired for **P2** and the new product **P2’’**, respectively. On Fig. 7B, for the **P2** structure, intracyclic fragments ^1,5^A_1_ at *m/z* 265.1, the corresponding water loss at *m/z* 247.1, and the fragment at *m/z* 221.1 which can be attributed to ^1,3^A_1_ or ^2,4^A_1_ (isobaric fragments) were observed. These fragments are missing for the new product. These indirect proofs lead to the structure **P2″** (glucosylation on OH-2_A_).

**﻿Figure 7:**
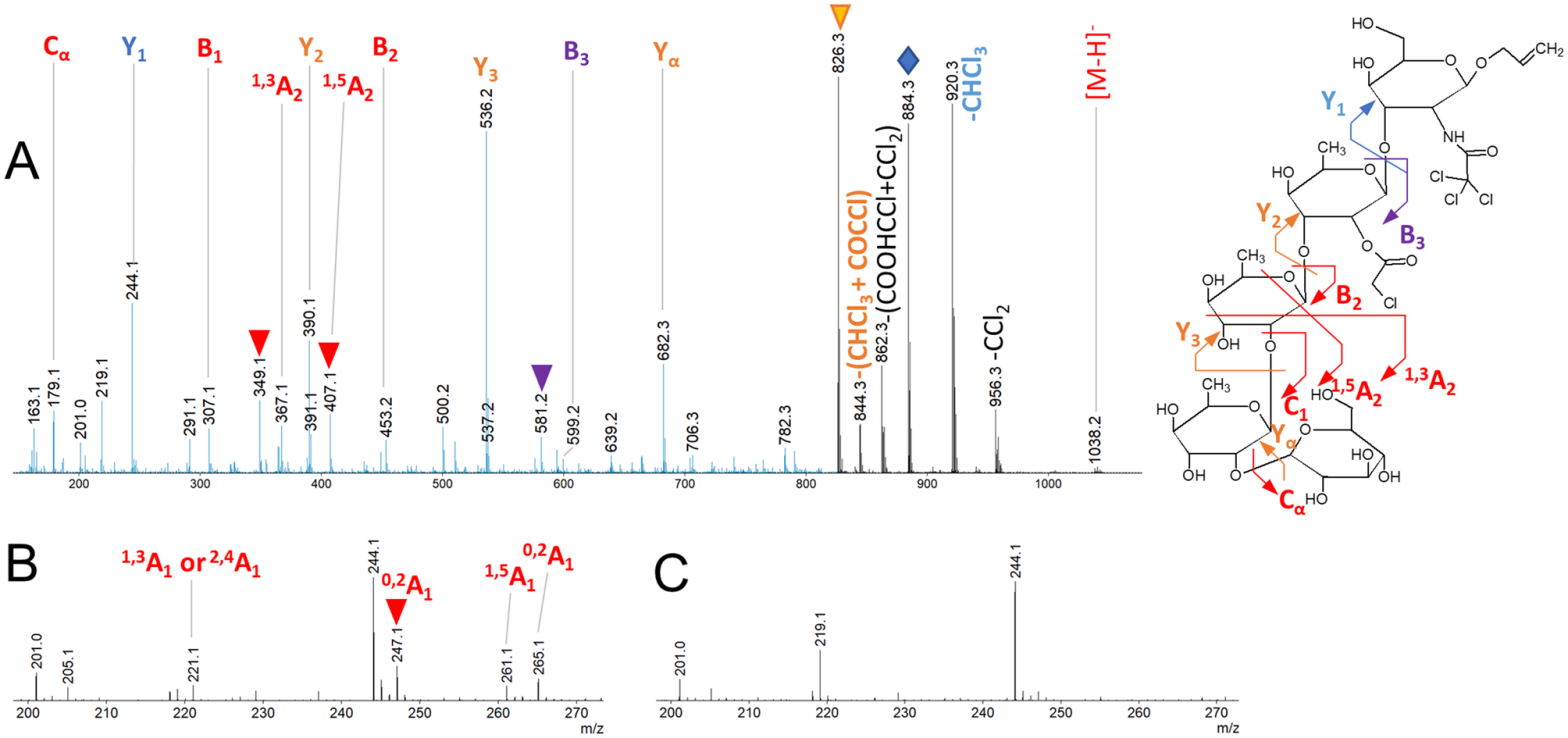
(**A**) UHPLC-ESI–MS/MS spectrum of the pentasacharide isolated as [M-H]^−^ at *m/z* 1038.21 at *t*_*R*_ = 22.07 min in samples M35 validated as being glucosylated on OH-2_A_. (**B**) Zoom in the mass range *m/z* 200–270 of the **P2** product (glucosylation on OH-3_A_). (**C**) Zoom in the mass range *m/z* 200–270 of the **P2″** product (glucosylation on OH-2_A_). The blue area of the spectrum is enlarged by a factor of 2 in the intensity axis. Annotations in red correspond to intact product ions. Annotation in blue correspond to product ions with a loss of CHCl_3_. Annotations in purple correspond to product ions with a loss of COCHCl. Annotation in orange correspond to product ions with a loss of CHCl_3_ + COHCl_3_. All these labile function losses are in agreement with the structure. Red * indicate consecutive fragmentations, ▽ indicate H_2_O loss, ◊ indicate HCl loss. For clarity, only one fragment per pair (B, C and Y, Z) was reported on the annotated structure.

**Figure 8:**
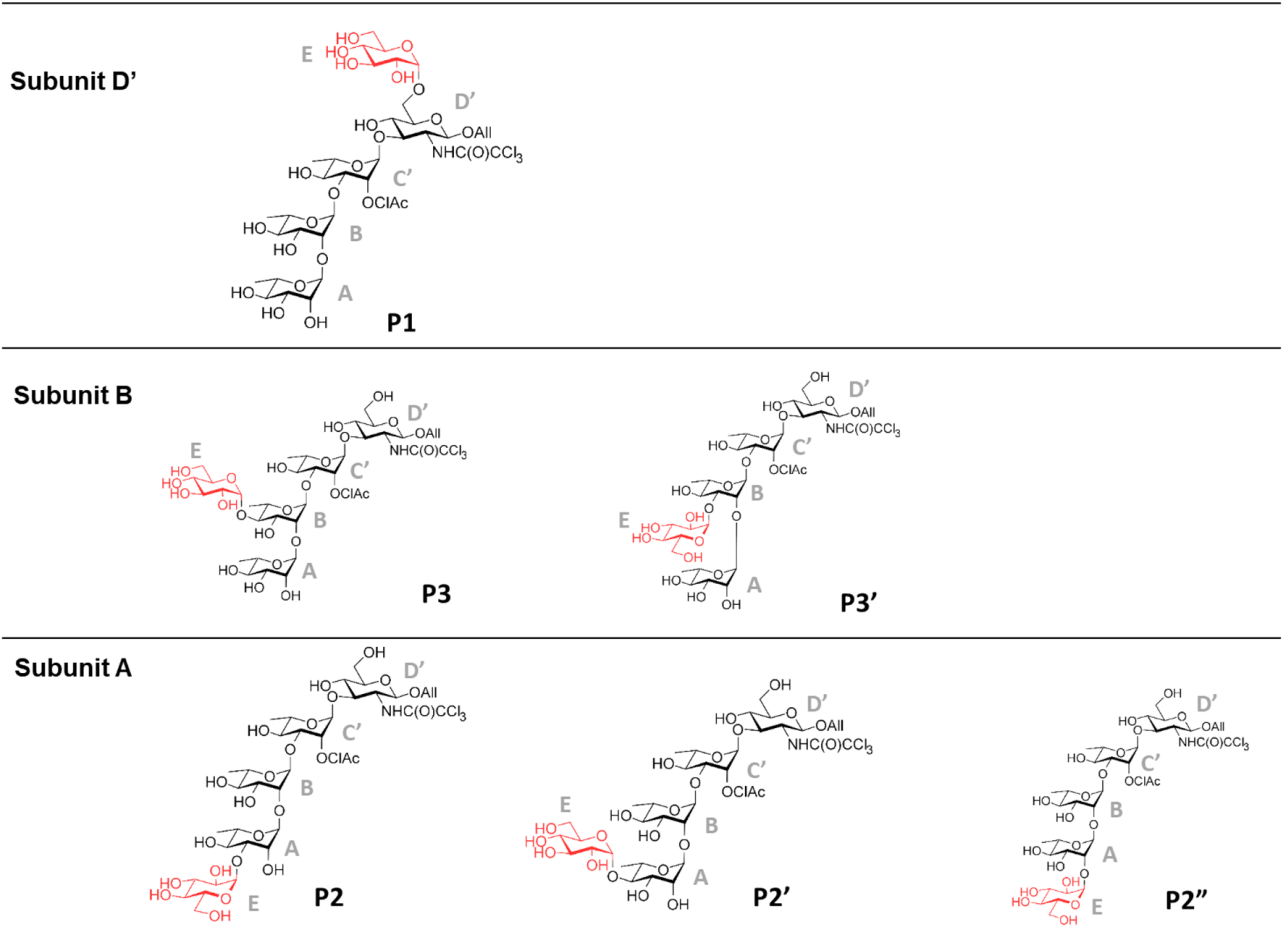
Diversity of pentasaccharides obtained with the BRS-B Δ2 mutants. The various products are shown on the basis of the glucosylated subunit (**A**, **B** or **D’**). **P1 ABC’[E(1 → 6)]D’**, characterized in ref ^24^. **P2 [E(1 → 3)]ABC’D’** and **P2’ [E(1 → 4)]ABC’D’** characterized in ref ^21^. **P3, P3’** and **P2″** were characterized in this study to be **A[E(1 → 4)]BC’D’** and **A[E(1 → 3)]BC’D’**, and **E[(1 → 2)]ABC’D’**, respectively. **P3** was characterized by NMR spectroscopy and **P3’** and **P2″** by MS/MS, respectively. ClAc: chloroacetyl. Drawing of chemical structures done using CHEMDRAW (PerkinElmer).

Finally, all the structures deciphered by the use of HPLC–UV, NMR and UHPLC-MS/MS are summarized in Fig. 8.

### Insight on the impact of mutations on product profiles

BRS-B Δ2 naturally favors glucosylation of the primary hydroxyl group OH-6_D’_ from **ABC’D’**. Group I comprising 16 mutants (M1 to M16) aimed at introducing beneficial mutations in order to obtain **ABC’[E(1 → 4)]D’** (Fig. 4A). Redesigning **D’** binding site required the introduction of 12–15 mutations to unclutter the active site and rend the hindered secondary OH-4_D’_ accessible to the glucosyl moiety. Such mutations turned out to have a highly detrimental impact on sucrose binding and cleavage, which could be explained by enzyme misfolding or stability loss. In the presence of **ABC’D’**, mutants from Group I kept the specificity of their parental enzyme, producing only pentasaccharides **P1** and **P2/P2’** but in different proportions. In spite of its 13 mutations and similarly to the parental enzyme, mutant M6 revealed a preference for **D’** subunit glucosylation to produce **P1**.

Group II encompassing 18 mutants (M17 to M34) aimed originally at glucosylating OH-4_C’_, the sole available hydroxyl function of **C’** moiety (Fig. 4C). Accessibility of this secondary hydroxyl group is highly hindered due to the constrained β-1,3 linkage between rhamnosyl residues **B** and **C’**, and the presence of the three protecting groups at **C’** and **D’** subunits. Introduction of 10–13 mutations were proposed by the design. These mutations affect sucrose recognition less severely as seven mutants were found relatively active toward sucrose and successfully glucosylated **ABC’D’**, producing up to four distinct pentasaccharides (**P1**, **P2**, **P2’** and **P3**) based on retention time. The versatility of these mutants to accommodate **ABC’D’** in different manners led to broader product specificity yielding novel molecular diversity (Fig. 6) but also resulted in a loss of selectivity (Fig. 5). Unlike the parental enzyme, most mutants were shown to glucosylate preferentially rhamnose **A**, forming **P2**/**P2’** glucosylated at OH-3_A_ and OH-4_A_. M28 and M31 even showed exclusive glucosylation of the **A** residue. Mutants M21, M23 and M30 revealed their ability for inner chain glucosylation at the **B** moiety, producing **P3**, which was never reported to be synthesized by native branching sucrases.

Group III gathers 15 mutants (M35 to M49) containing between 9 and 12 mutations aiming at favoring end chain glucosylation, targeting OH-3_A_ or OH-4_A_ to improve the production of **P2/P2’**, only weakly achieved by parental enzyme (Fig. 4D). Here again, the introduced mutations turned out to drastically affect sucrose recognition. However, all three mutants (M35, M40 and M41) revealed their ability to form, in addition to **P1** and **P2/P2’,** a new product not synthesized by the parental enzyme. Glucosylation of OH-2_A_ was observed for the first time with mutant M35, yielding pentasaccharide **P2**″ and enabling glucosylation of the third hydroxyl group from the **A** moiety, although of less interest in the *S. flexneri* context as this position is involved in chain elongation. Mutants M40 and M41 enabled glucosylation of the inner chain **B** rhamnose, providing respectively access to **P3’** (glucosylated at OH-3_B_) and **P3** (glucosylated at OH-4_B_). Here again, with these mutants, we gained access to all possible glucosylation patterns of the **B** moiety.

Computational protein design undertaken here was highly challenging as four subsites had to be re-designed to improve tetrasaccharide **ABC’D’** accommodation for each targeted pentasaccharide without losing the affinity for sucrose. The huge combinatorial sequence corresponding to the 27 selected positions of mutations was drastically reduced thanks to the design. By screening a very limited set of 49 sequences, containing a high number of mutations (between 9 and 15 depending on the design), several mutants were successfully isolated. Impressively, after a challenging structural analysis of the products, we found out that these mutants enabled the glucosylation of six out of the eight hydroxyl groups of the lightly protected **ABC’D’** acceptor substrate. It is noteworthy that three of the resulting pentasaccharides were characteristic of *S. flexneri* type-specific O-Ags (4a/4b, 5a and 3a) for which no enzymatic route has been proposed yet. Remarkably, two mutants (M40 and M41) showed a product profile in line with the design expectations. Experimental deconvolution of the mutations in these mutants could help to better understand the contribution of each mutation and their combinatorial effect.

These results highlight the difficulties of redesigning an active site as large and exposed as that of branching sucrases for the selective glucosylation of a structurally complex and chemically modified molecule, presenting no similarity with the natural substrate. This required introduction of a large charge of amino acid mutations to target glucosylation at various hydroxyl positions but also made it difficult to control the enzyme selectivity to produce a single pentasaccharide. This effect was further pronounced due to the exposure and flexibility of the active site. Furthermore, re-engineering enzymes catalyzing multi-step reactions from multiple substrates considerably enhanced complexity of the design, requiring multi-objective optimization *i.e.* in our case the ability to utilize sucrose donor and novel specificity toward an unnatural acceptor.

Another limitation in the computational redesign was the lack of crystallographic structure that led us to assume that reliable 3D-modelling could be performed given the high sequence identity between BRS-B Δ2 and the sole GH70 branching sucrase of known structure to date (ΔN_123_-GBD-CD2). Nonetheless, the suggested flexibility of several loops surrounding the active site^36^ could drastically alter topology of the active site and recognition of acceptor **ABC’D’**. Undoubtedly, accuracy of the design would have benefited from the determination of crystallographic structures of the enzyme in complex with the acceptor or with the products. However, a successful outcome of the design would still not be warranted given the many limitations still faced by computational protein design methods such as the poor integration of molecular flexibility and conformational rearrangements^21^, the under consideration in energy functions of entropy penalty and solvent effect, etc.

## Conclusion

Computer-aided design was for the first time applied to the redesign of several acceptor subsites of a branching sucrase to redirect glucosylation regioselectivity toward various hydroxyl functions of a lightly protected tetrasaccharide **ABC’D’**, designed as a suitable precursor in the synthesis of serotype-specific *S. flexneri* haptens. By predicting the introduction of as many as 15 amino acid mutations in the active site, mutants were found to be able to produce up to six distinct pentasaccharides, whereas only two were synthesized by the parental enzyme. As discussed in the manuscript, the use of highly sensitive UHPLC-MS method unveiled the presence of a higher structural diversity in the reaction medium than with conventional methods. This opens new venues for emerging mass spectrometry approach that use high resolution Ion mobility^43^ to unambiguously characterize molecular structures present in complex carbohydrate mixes. Herein, we demonstrated for the first time that mutants could perform branching reactions onto **ABC’D’**. This remarkable achievement could advantageously open the way to the glucosylation of longer oligosaccharide chains, such as fragments of the O-Ag backbone shared by most *S. flexneri* serotypes. Given that no equivalent enzymatic activity has been identified in Nature, to our knowledge, these mutants are promising starting templates for further rounds of evolution and/or optimization. Yet, fine tuning of this enzyme’s active site is still difficult due to the lack of detailed understanding of the reaction mechanism and 3D structural information. Although objectives of the computational design were not fully met, the impressive malleability of the acceptor binding site and the demonstrated ability to expand the tolerated productive binding modes for the **ABC’D’** acceptor still led to unique and exciting achievements that could offer novel opportunities for the development of highly convergent chemo-enzymatic routes toward *S. flexneri* haptens.

## Material and methods

### Chemical synthesis of the tetrasaccharide ABC’D’

The synthesis of the tetrasaccharide **ABC’D’** was performed as described^24^.

### Computational enzyme design

The 3D model of BRS-B Δ2 was constructed by comparative modelling using MODELLER software^37^, using the reference coordinates of the template ΔN_123_-GBD-CD2 (PDB ID: 3TTQ, resolution 1.9 Ǻ)^38^. The same strategy as described in our previous study of the complexes ΔN_123_-GBD-CD2:pentasaccharides was used for docking the **ABC’[E(1 → 4)]D’**, **AB[E(1 → 4)]C’D’** and **[E(1 → 3)]ABC’D’** pentasaccharides in the active site and for the calculation of the force field parameters^21^. Between 12 and 16 designable positions were selected for the redesign based on our intuition after careful visual inspection of the enzyme:pentasaccharide interfaces using PYMOL 1.7 (PyMOL Molecular Graphics System, Schrödinger, LLC)^39^. This graphical examination was helped by the MMGBSA calculations performed to evaluate the individual amino acid contributions to the free energy binding of BRS-B:pentasaccharide for each system and described in details in the Supporting Information. The residues having Cα within 10 Å of the Cα of the redesignable residues were allowed to be repacked. 20,000 independent runs were carried out for each system using the RosettaDesign ^30^ software and Beta_Nov15 energy function ^40^. The output sequences were filtered based on different Rosetta scoring and filtering schemes. The RosettaDesign protocol and sequence filtering are described in deep details in the Supporting Information and Methods.

### Mutant construction

Plasmid pET55-BRS-B_Δ2_WT was obtained after 5’ truncation of 459 base pairs in the gene *brsB* (Genbank accession number CDX65123.1)^31^ from *Leuconostoc citreum* NRRL B-742 using the method described by Wang and Malcom^41^. This truncation resulted in the enzyme BRS-B $\Delta$2, showing the same expression levels and activity as BRSB and BRS-B $\Delta$1. Briefly, the method comprised the use of a two-stage procedure, based on the QuickChange Site-directed Mutagenesis protocol (Stratagene, La Jolla, CA, USA) with a pre-PCR consisting in a single-primer extension stage before the standard protocol and allowing deletion of the sequence of interest. The PCR product was cloned into pET55 plasmid using Gateway technology (Thermo Fisher Scientific) for expression in *E. coli* under the control of T7 promoter and with a gene for ampicillin resistance. Plasmid pET55-BRS-B_Δ2_E709Q was obtained after performing E709Q mutation in *brsB* gene. Gene *brsB* was amplified by inverse PCR using forward primer CAT-ATT-TCA-ATT-GTT-**CAG**-GCT-CCA-AAG-GGG-GAA-AC and reverse primer TAT-GTT-GAT-TGG-CAA-CTG-CCT-CAT-TGT-CAG. Parental plasmid was digested by DpnI enzyme (NEB) and ligation was performed using T4 DNA ligase (NEB). The reaction was purified using NucleoSpin PCR Clean-up (Macherey–Nagel) before transformation into *E. coli* TOP10 (Invitrogen). Correct sequence was verified by Sanger sequencing (GATC Biotech). Plasmids pET55-BRS-B_Δ2_M1 to pET55-BRS-B_ Δ2_M49 were purchased from GenScript.

### Production of a library of 49 BRS-B Δ2 mutants

Plasmids pTf16 containing chaperone gene *tig* under the control of promoter *araB*, and pG-KJE8 containing chaperone genes *dnaK, dnaJ, grpE, groES, groEL* under the control of promoters *araB* and *Pzt-1* (Takara) were chemically transformed into *E. coli* BL21 Star (DE3) (Life Technologies). Strains were rendered competent again and chemically transformed using plasmids pET55-BRS-B_ Δ2_WT, pET55-BRS-B_Δ2_E709Q and pET55-BRS-B_Δ2_M1 to pET55-BRS-B_ Δ2_M49 encoding for parental BRS-B Δ2, inactive mutant of BRS-B Δ2 and the 49 BRS-B Δ2 mutants, respectively. After overnight preculture at 37 °C in LB medium (10 g L^−1^ tryptone, 5 g L^−1^ yeast extract, 10 g L^−1^ NaCl) containing 100 µg.mL^−1^ ampicilin (Euromedex) and 12.5 µg.mL^−1^ chloramphenicol (Sigma), the main culture was inoculated at optical density at 600 nm (OD_600nm_) = 0.05 and used (i) modified ZYM-5052 medium with 0.1% d-lactose, 1% glycerol^24,42^, (ii) 2 × YT medium (20 g L^−1^ tryptone, 10 g L^−1^ yeast extract, 10 g L^−1^ NaCl) or (iii) optimized ZYM-5052 with 0.05% d-glucose, 0.75% d-lactose, 1.5% glycerol ^34^. Medium were supplemented with 100 µg.mL^−1^ ampicillin and 12.5 µg.mL^−1^ chloramphenicol as selection markers. When using medium (ii) and (iii), chaperone protein expression was induced immediately after inoculation by adding 4 g L^−1^ of l-arabinose (ACROS Organics) for pTf16 or 4 g L^−1^ of l-arabinose and 10 ng.mL^−1^ tetracycline (Sigma) for pG-KJE8. Cultures (i) and (iii) were grown at 21 °C in either 50 mL, 200 mL or 1 L scale in baffled flasks for 24 h (pTf16) or 32 h (pG-KJE8). When using medium (ii), production of the recombinant enzymes was induced using isopropyl β-d-1-thiogalactopyranoside (IPTG) when OD_600nm_ reached 0.4 after growth at 37 °C, and the temperature was lowered to 21 °C for additional 24 h culture. Cells were harvested by centrifugation and re-suspended in appropriate volumes of Sodium acetate (NaOAc) buffer 50 mM pH 5.75 to concentrate the cells at OD_600nm_ = 15. Cells were then disrupted by sonication and the cellular debris were removed by centrifugation at 13,000 g for 30 min at 8 °C to recover the soluble fraction. Both insoluble and soluble fractions were analyzed using SDS-PAGE to assess the production of the enzymes.

### Library screening on sucrose in liquid DNS-based ON/OFF assays

The hydrolytic activity of the enzymatic crude extracts on sucrose (100 g L^−1^) was assessed at 30 °C in acetate sodium buffer 50 mM pH 5.75. After 70 h incubation, 100 µL of the reaction mixtures were mixed with the same volume of DNS (3,5-dinitrosalicylic acid) solution^43^ and heated to 95 °C for 5 min. Absorbance was read at a wavelength of 540 nm and active enzymes were selected by comparing the absorbance value with the one from inactive mutant E709Q that constituted the baseline.

### Purification and concentration of the best mutants

Mutants active on sucrose were produced as previously described with the following exception: after sonication the soluble fraction was recovered by centrifugation at 50,000 g (instead of 13,000 g) for 30 min at 8 °C. Purification was performed as previously described^21,24^. The mutant enzymes were then concentrated using AmiconUltra-15 (Merck Millipore) with a cutoff of 50 kDa. Concentration was possible to different extents before aggregation depending on the mutant. The final concentrations were measured using Nanodrop instrument (Thermo Scientific) and considering the theoretical molecular weight (MW = 132 480 g mol^−1^) and molar extinction coefficient (ε = 196 770 M^−1^.cm^−1^) of BRS-B Δ2 calculated with ProtParam tool from ExPASy server. Concentrations were 12.5 g L^−1^ for M6, 1.3 g L^−1^ for M14, 3.1 g L^−1^ for M18, 2.9 g L^−1^ for M21, 7.1 g L^−1^ for M23 and 3.9 g L^−1^ for M30, 0.2 g L^−1^ for M28, 0.2 g L^−1^ for M31, 3.7 g L^−1^ for M34, 0.7 g L^−1^ for M35, 1.9 g L^−1^ for M40 and 1.0 g L^−1^ for M41.

### Specific activity determination on sucrose

The specific activities of mutants M21, M23, M30, M34 and M35 were determined in 200 µL assays in microtiter plates at 30 °C in sodium acetate buffer 50 mM pH 5.75 over the course of 50 min, using 100 g L^−1^ of sucrose and purified concentrated enzymes in various amounts (see Purification and concentration of the best mutants)0.30 µL sampling was performed every 10 min.

### Reaction assays with sucrose and ABC’D’ acceptor

Reactions were performed in presence of the tetrasaccharide acceptor **ABC’D’** (50 mM) and sucrose (1 M) in miniaturized assay at 50 µL scale, in NaOAc buffer 50 mM pH 5.75, using various purified enzymes. The amount of enzymes depended on the mutant. After 16 h incubation, reactions were stopped using a 30% aqueous solution of acetonitrile supplemented with 0.08% TFA.

### HPLC–UV-MS for tetrasaccharide and product profiles

The same analytical methods as previously described were used^21^. Briefly, 10 µL of the carbohydrate mixtures were injected in HPLC (Dionex UltiMate 3000, Thermo Scientific, San Jose, CA, USA) and separated at 1 mL.min^−1^ onto a C18-RP Fusion column (4 µm, 80 Å, 250 × 4.6 mm) kept at 40 °C by using the following method (with A: pure water and B: pure acetonitrile): an isocratic step of A/B (80:20, v/v) for 15 min followed by a linear gradient to A/B (20:80, v/v) in 15 min. After maintaining this ratio for 5 min, the column was re-equilibrated at A/B (80:20, v/v) for 11 min.

Detection was performed by UV at a wavelength of 220 nm and the system was coupled to a LTQ Orbitrap Velos hybrid ion trap-orbitrap mass spectrometer (Thermo Fisher Scientific, San Jose, CA, USA).

### Determination of pentasaccharide structures

#### NMR experiments

For NMR studies, samples were lyophilized three times and dissolved in 180 µL of 99.9% DCl-containing D_2_O pH 5.1. All NMR spectra were recorded on a Bruker Avance spectrometer operating at a proton frequency of 950 MHz (TGIR- RMN-THC Fr3050 CNRS, Gif-sur-Yvette) and at a carbon frequency of 238 MHz with a 5-mm gradient indirect cryoprobe. All spectra were processed and analyzed with the Topspin software (Bruker) and Sparky software (T. D. Goddard and D. G. Kneller, SPARKY 3, University of California, San Francisco).

^1^H and ^13^C NMR spectra were accumulated at 25 °C, 65,536 data points were acquired with 32 and 2048 scans respectively for proton and carbon experiments. ^1^H-^13^C HSQC (Heteronuclear Single Quantum Coherence spectroscopy) and Double Quantum Filtered COrrelation SpectroscopY (QDF COSY)^44^ experiments were performed at 25 °C. Homo and heteronuclear spectra were recorded under the following experimental conditions: 512 increments of 2048 complex points are acquired with an accumulation of 16 scans. Spectral widths were 16,025 Hz for proton dimension and 44,267 Hz for carbon dimension.

#### UHPLC MS/MS characterization

MS/MS studies were performed on the BIBS facility at INRAE Nantes. Analyses were carried out on a UHPLC-ESI-Q-Tof platform composed of an Acquity UPLC H-class system, coupled to a Synapt G2Si HD mass spectrometer (Waters Corp., Manchester, UK). Reaction media were diluted 250 times in H_2_O/acetonitrile (95.5:4.5, v/v). The separation of the glucosylated products was performed by LC using a Porous Graphitized Carbon (PGC) (Hypercarb (2.1 mm × 100 mm, 3 µm)) analytical column placed in an oven at 80 °C. The injected sample amount was 10 µL and the flow rate was set to 400 µL.min^−1^. A binary gradient was used (A: pure water, B: pure acetonitrile): from 2 to 25% of solvent B in 10 min, then up to 73% at 23.5 min and maintained at 73% for 4 min.

Mass detection was carried out with the instrument operating in negative polarity over mass ranges of *m/z* 350–2000 in MS mode and *m/z* 150−1100 in MS/MS mode. The spray voltage was set at 3.5 kV. MS/MS analysis in collision-induced dissociation were performed by selected the mass of the precursors of interest as [M-H]^−^ at m/z 1038.21 in the quadrupole prior to their fragmentation in the transfer cell of the instrument (collision energy adjusted at 30 V). Argon was used as the collision gas. Data acquisition was carried out using the MassLynx software (V4.1). Data were converted into mzml format using MSConvert^45^ and processed with Mmass 5.5.0^46^. Annotations of spectra and structures were performed according to the nomenclature of Domon and Costello^47^.

## Supplementary Information

Supplementary Information.

## Data Availability

All the data generated and analyzed is available in this published article or in its supplementary information. This available data may be requested from the corresponding author.
