# Supplementary material for: Computer-aided engineering of a branching sucrase for the glucodiversification of a tetrasaccharide precursor of S. flexneri antigenic oligosaccharides
Source: Sci Rep. 2021 Oct 13;11:20294. doi: 10.1038/s41598-021-99384-9 (PMC8514537; doi:10.1038/s41598-021-99384-9)
Supplement: Supplementary file 1 — Supplementary Information. [file 41598_2021_99384_MOESM1_ESM.docx]

**Supplementary Information**

## **Computer-aided engineering of a branching sucrase for the glucodiversification of a tetrasaccharide precursor of *S. flexneri* antigenic oligosaccharides**

Mounir Benkoulouche^1#^, Akli Ben Imeddourene^1#^, Louis-Antoine Barel^2,3^, Dorian Lefebvre^1^, Mathieu Fanuel^4,5^, Hélène Rogniaux^4,5^, David Ropartz^4,5^, Sophie Barbe^1^, David Guieysse^1^, Laurence A. Mulard^2^, Magali Remaud-Siméon^1^, Claire Moulis^1^ and Isabelle André^1^*

*^1^ Toulouse Biotechnology Institute, TBI, Université de Toulouse, CNRS, INRAE, INSA, Toulouse, France. 135, avenue de Rangueil, F-31077 Toulouse Cedex 04, France
^2^ Unité de Chimie des Biomolécules, Institut Pasteur, UMR3523 CNRS, 28 rue du Dr Roux, 75724 Paris Cedex 15, France*

*^3^ Université Paris Descartes, Sorbonne Paris Cité, Paris, France*

*^4^ INRAE, UR BIA, F-44316 Nantes, France*

*^5^ INRAE, BIBS Facility, F-44316 Nantes, France*

# Both authors contributed equally

* Corresponding author: isabelle.andre@insa-toulouse.fr

### **Supporting Computational Methods**

**MMGBSA calculation**

In order to estimate per residue enthalpy contribution to the enzyme:pentasaccharide free energy binding, the Molecular Mechanics (MM) energies combined with the Generalized Born (GB) and Surface Area (SA) continuum solvation calculation were carried out on enzyme-pentasaccharide complexes, using MMPBSA.py.MPI module of AMBER program. The generalized Born calculation was performed with the model developed by the Case group^1^ using igb=5 control command and a salt concentration of 0.1 M. The surface area for the nonpolar solvation term was calculated using Linear Combination of Pairwise Overlaps algorithm^2^, and the surface tension was set to default value *i.e*. 0.0072 kcal/mol·Å².

**Shannon entropy calculation**

Position-dependent amino acid residue variation in available GH70 sequences aligned by CLUSTALW, were analyzed using the Shannon information entropy measure (*H_X_*), calculated using our in house SEQUESTER software ^3^.

#### **Rosetta Design protocol**

The Rosetta python script molefile_to_params.py was used to convert mol2 files to Rosetta parameter files and to generate ligands (sucrose and pentasaccharides) coordinates fitting the Rosetta atom names. Before the design, the enzyme and pentasaccharide interactions were optimized by a gradient-based minimization without restraints on the ligands. After that, 3 cycles of sequence design/packing and minimization were performed. Throughout the packing steps, the soft-repulsive force field was used, the input structure coordinates was included in the rotamer set, and the sub-rotamer Chi1 and Chi2 were taken into account. During all the computational design protocol steps (minimization/design/packing) the flexibility of the backbone and the side chain dihedrals of designable/repackable region was allowed, while the catalytic residues were prevented from being repacked or minimized.

#### **Output Sequences filtering**

In order to compare the parental enzyme and the designed sequences scores, the parental enzyme:pentasaccharide complexes (starting points of the computational design) were scored using the same steps of interface optimization and 3 cycles of repack minimization except for the catalytic residues. Then, the first sequences selection was performed by comparing the total and enzyme-ligand interface scores of the mutants to parental enzyme total and interface scores (Figure S2). The second round of data processing was the enzyme:sucrose complexes scoring, after the docking of the sucrose in the active site of the mutants having total and interface scores less than the parental enzyme. The docking was carried out by taking the coordinates of the glucosyl moiety of the pentasaccharides for sucrose glucosyl unit. Afterwards the enzyme:sucrose complexes were scored by Rosetta by the same protocol used for the parental enzyme:pentasaccharides and described above. The resulting sequences were then clustered by the *Decrease Redundancy* module of *ExPASy* webserver^4^. The best score sequences of each cluster was selected for experimental testing (Table S1).

### **Supporting Figures**


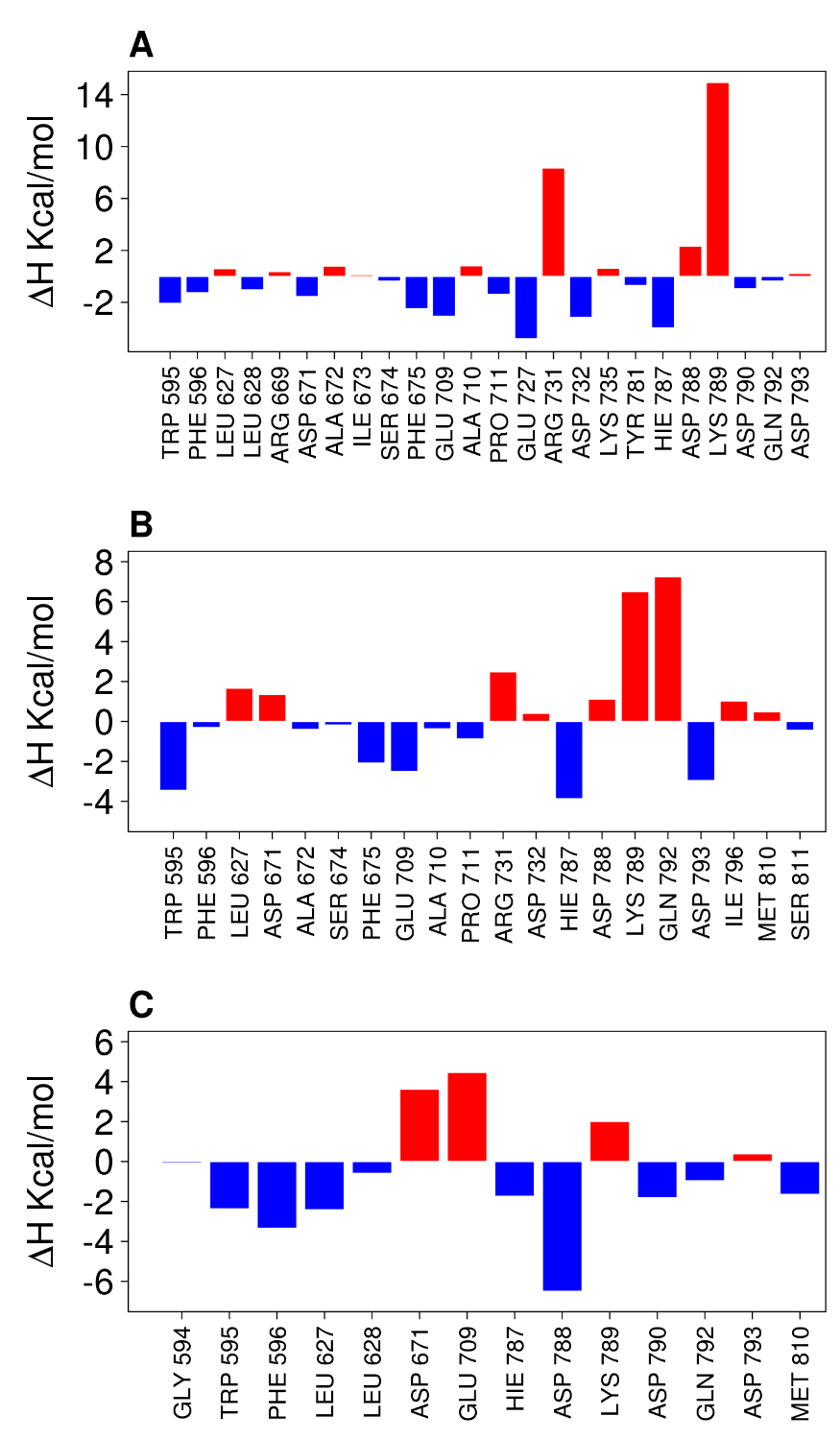


**Figure S1:** Graphics representing the amino acid residue enthalpy contribution to the free energy binding of the BRS-B Δ2 and **ABC’[E(1→4)]D’** (*S. flexneri* 1a/1b) (A), **AB[E(1→4)]C’D’** (*S. flexneri* 2a) (B) and **E(1→3)ABC’D’** (*S. flexneri* 3a) (C) complexes. The enthalpies were calculated from the redesign starting point of each system, the negative and positive values are represented by blue and red colors.


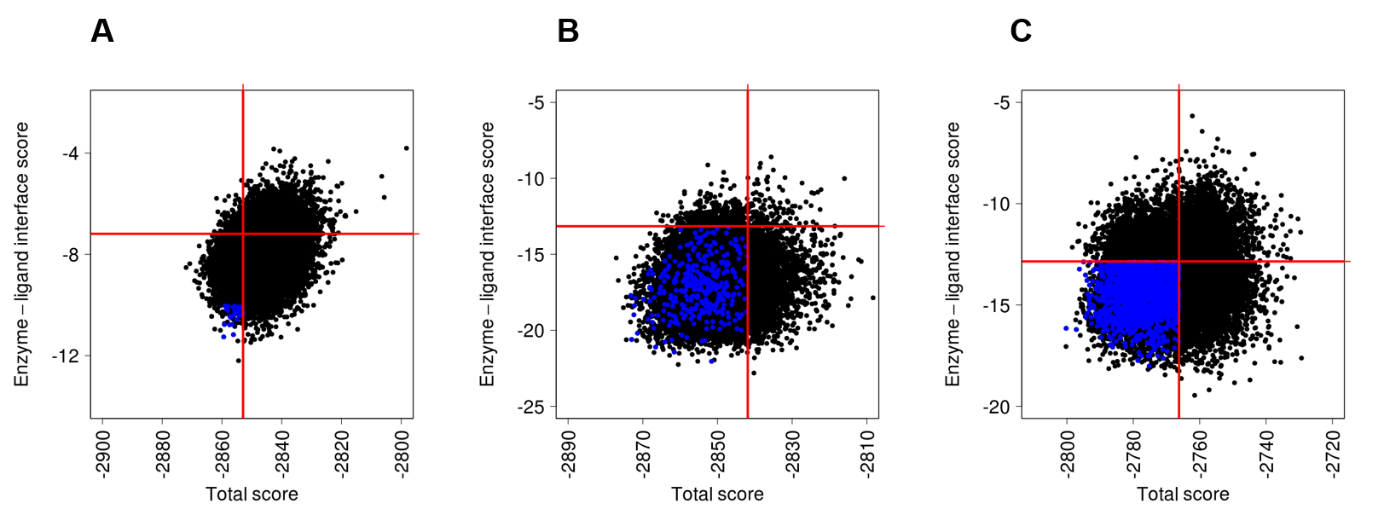


**Figure S2**: Graphics representing the total score (x-axis) against the enzyme:pentasaccharide interface score (y-axis) of the BRS-B:**ABC’[E(1→4)]D’** (A), BRS-B:**AB[E(1→4)]C’D’** (B) and BRS-B : **[E(1→3)]ABC’D’** (C) complexes represented in black solid circles and their enzyme:sucrose complexes from the first selection described in the text are illustrated by blue solid circles. The starting parental complex (BRS-B Δ2) total score and enzyme:pentasaccharide interface score are illustrated by the red lines.


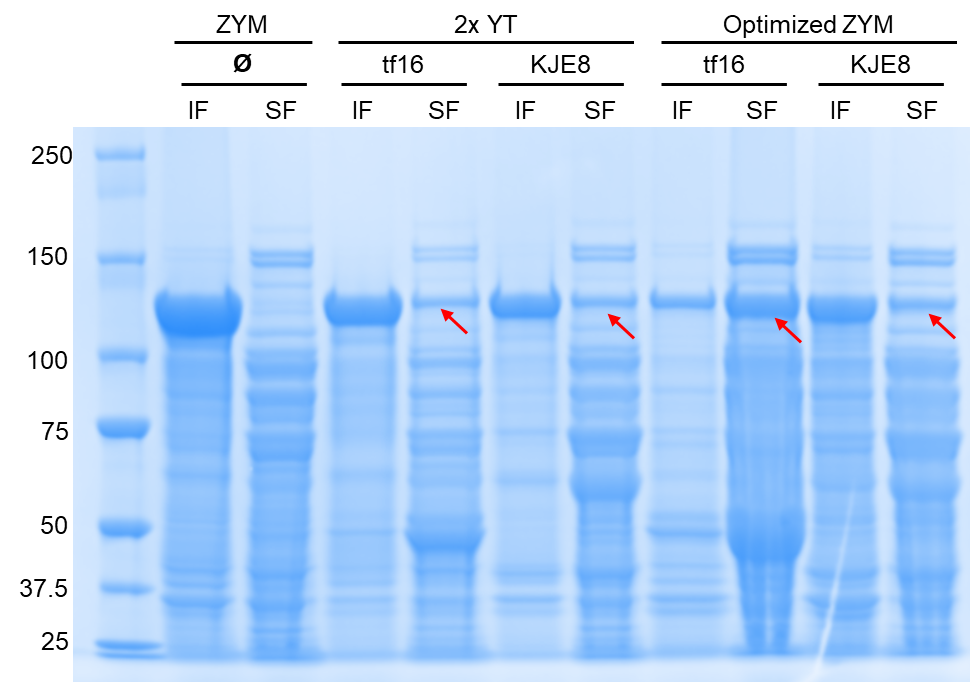


**Figure S3**: Optimization of the soluble expression of mutant M21. Size marker is present in lane 1 (sizes indicated in kDa). Using ZYM medium and without chaperone proteins (Ø), all the enzyme was produced in the insoluble fraction (IF, lane 2), no band was detected at the expected size of 132 kDa in the soluble fraction (SF, lane 3). When chaperone proteins (tf16 or KJE8) were co-expressed with M21 in 2x YT medium (lane 4 to 7), a band was detected at the expected size in soluble fractions (red arrows). The size of the band was further increased after optimization of the ZYM medium (lanes 8 to 11), especially when using tf16 chaperone proteins in the present case.


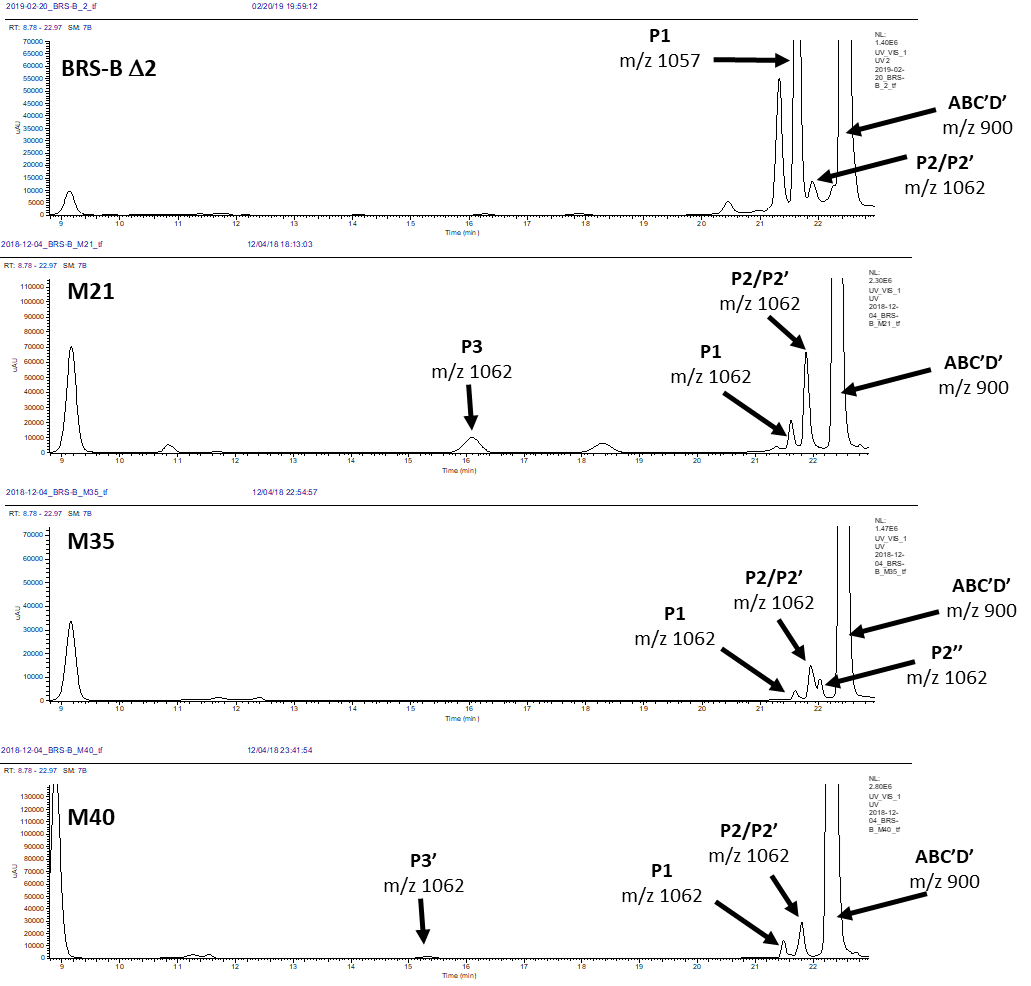


**Figure S4**: HPLC-UV220nm and MS analysis of glucosylation products obtained with BRS-B Δ2 and mutants M21, M35 and M40 that produced the 6 distinct pentasaccharide products (**P1** (*t_R_* = 21.6 min)**, P2** (*t_R_* = 21.9 min)**, P2’** (*t_R_* = 21.9 min)**, P2”** (*t_R_* = 22.1 min), **P3** (*t_R_* = 16.3 min)**, P3’**( *t_R_* = 15.3 min) ), after 16 h of reaction in presence of sucrose (1 M) and tetrasaccharide **ABC’D’** (50 mM) at pH 5.75 and 30°C. The *m/z* detected by MS is indicated for each product and corresponding to Na^+^ (1062) and NH4^+^ (1057) adducts in positive mode.


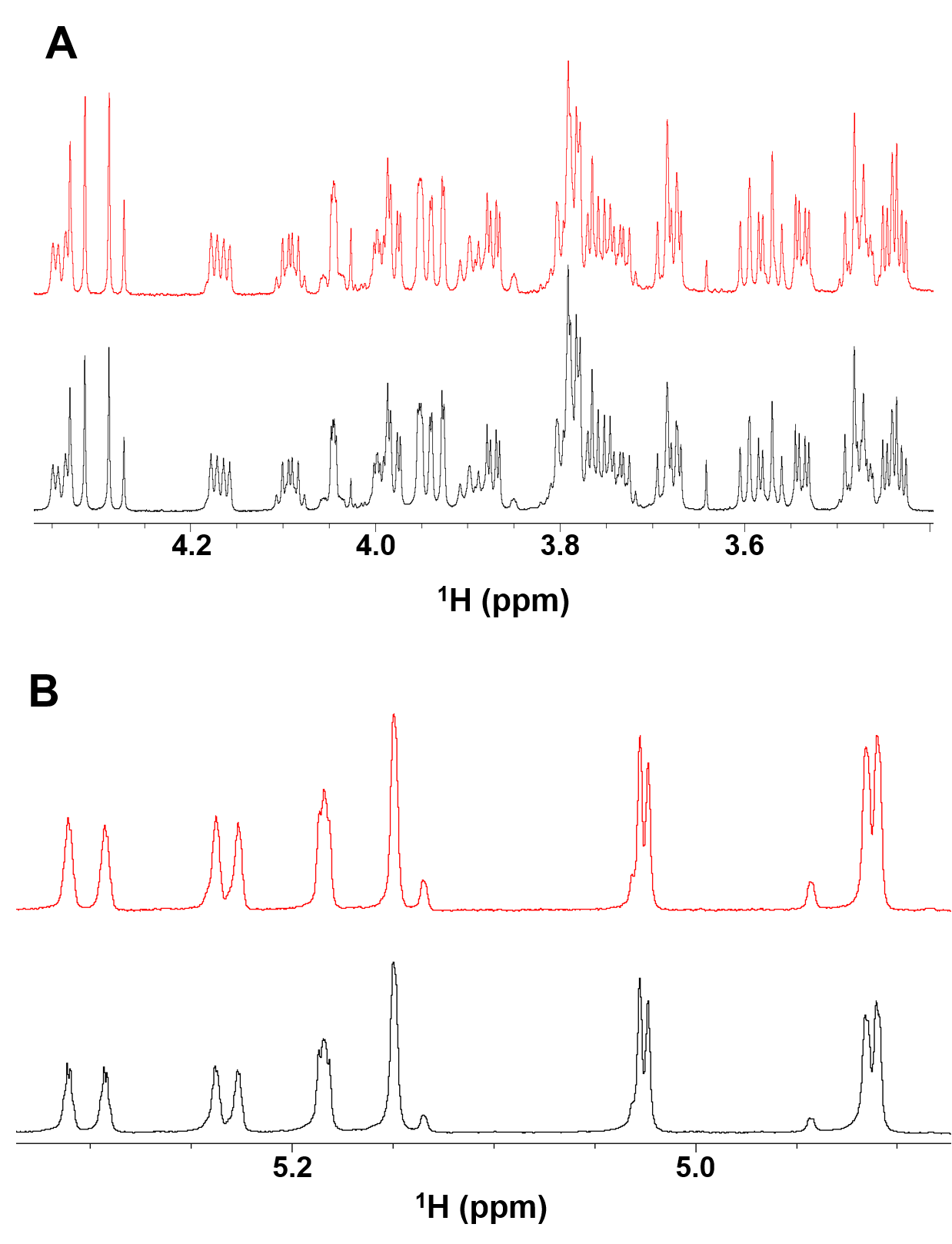


**Figure S5**: 1D spectra of the H2, H3, H4 and H5 region (A) and the anomeric region (B) of the pentasaccharides **P2’** obtained by the F2163G of ΔN_123_-GBD-CD2 (black) and by the M21 of BRS-B Δ2 at 25°C in D_2_O.

**
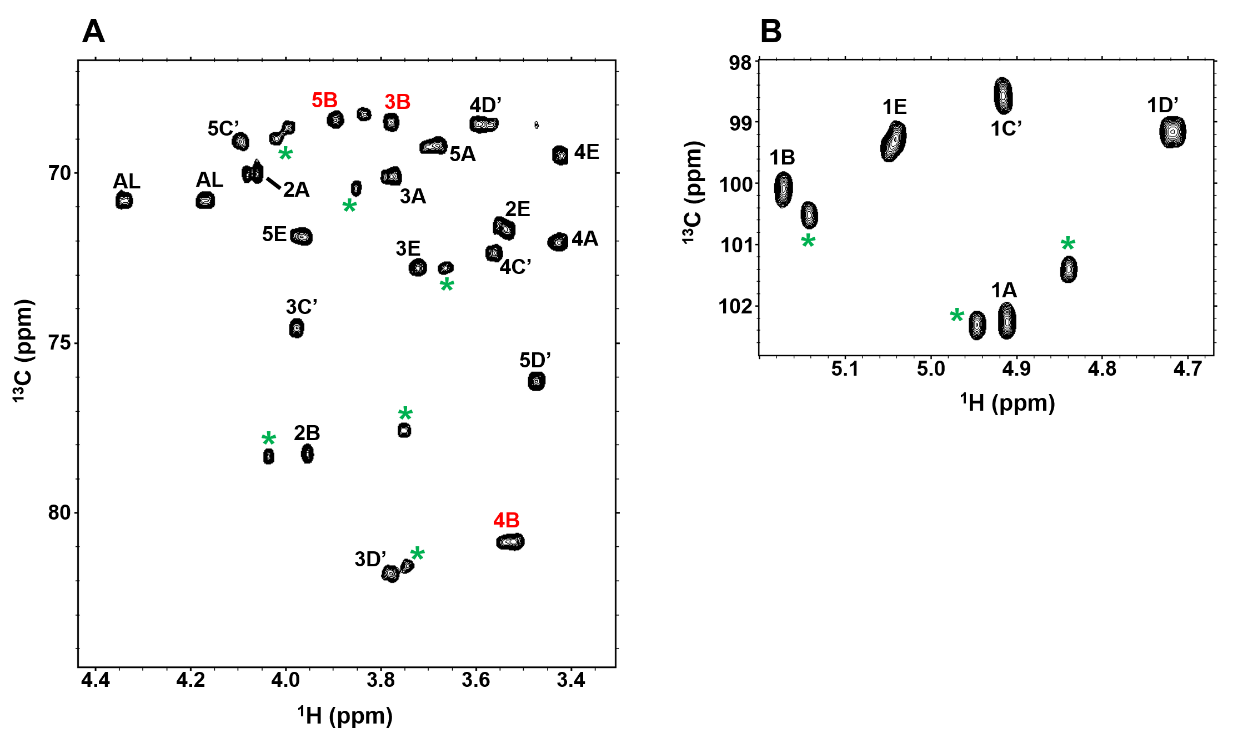
**

**Figure S6:** ^1^H-^13^C HSQC spectra of the C2, C3, C4 and C5 resonances (A) and the anomeric region (B) of the mixture **P3** and its dechloroacetyled form. The shifted resonances of the **P3**, compared to the tetrasaccharide, were labeled in red. The peaks of the dechloroacetyled form **ABCD’** are labelled by green stars. The spectrum was acquired at 950MHz.


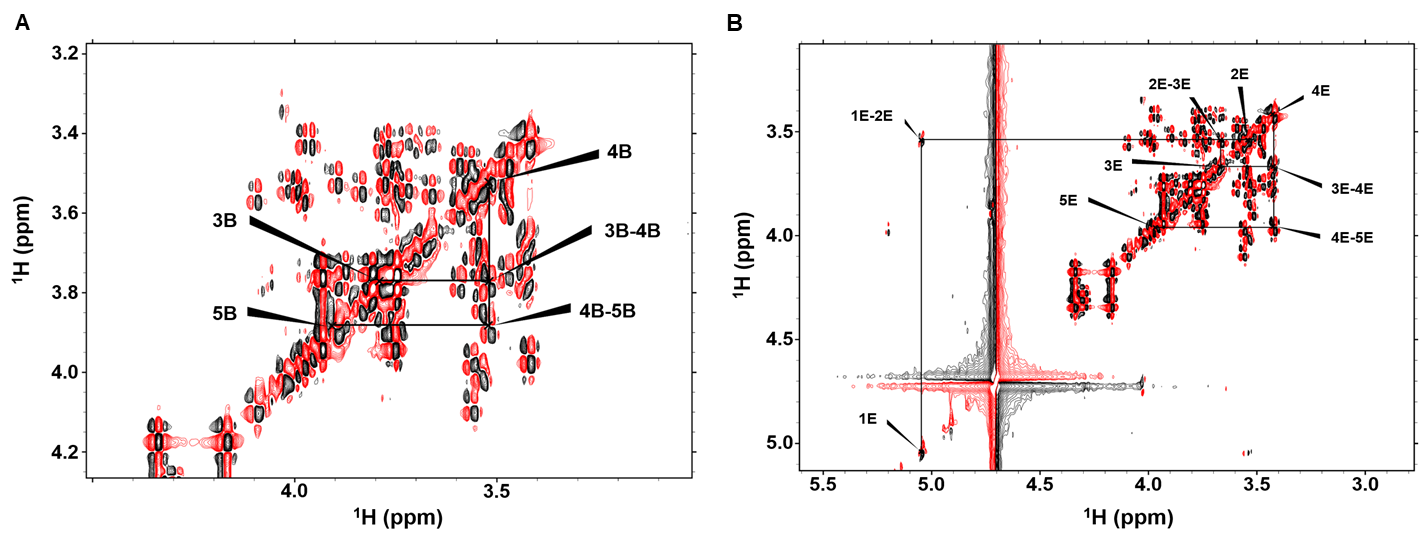


**Figure S7:** ^1^H-^1^H QDF-COSY spectra at 950MHz of the mixture of the **P3** and its dechloroacetyled form. Positive and negative peaks are showed in black and red respectively. The B (panel A) and E (panel B) units belonging to the **P3** connectivities are plotted and the corresponding peaks are labeled.


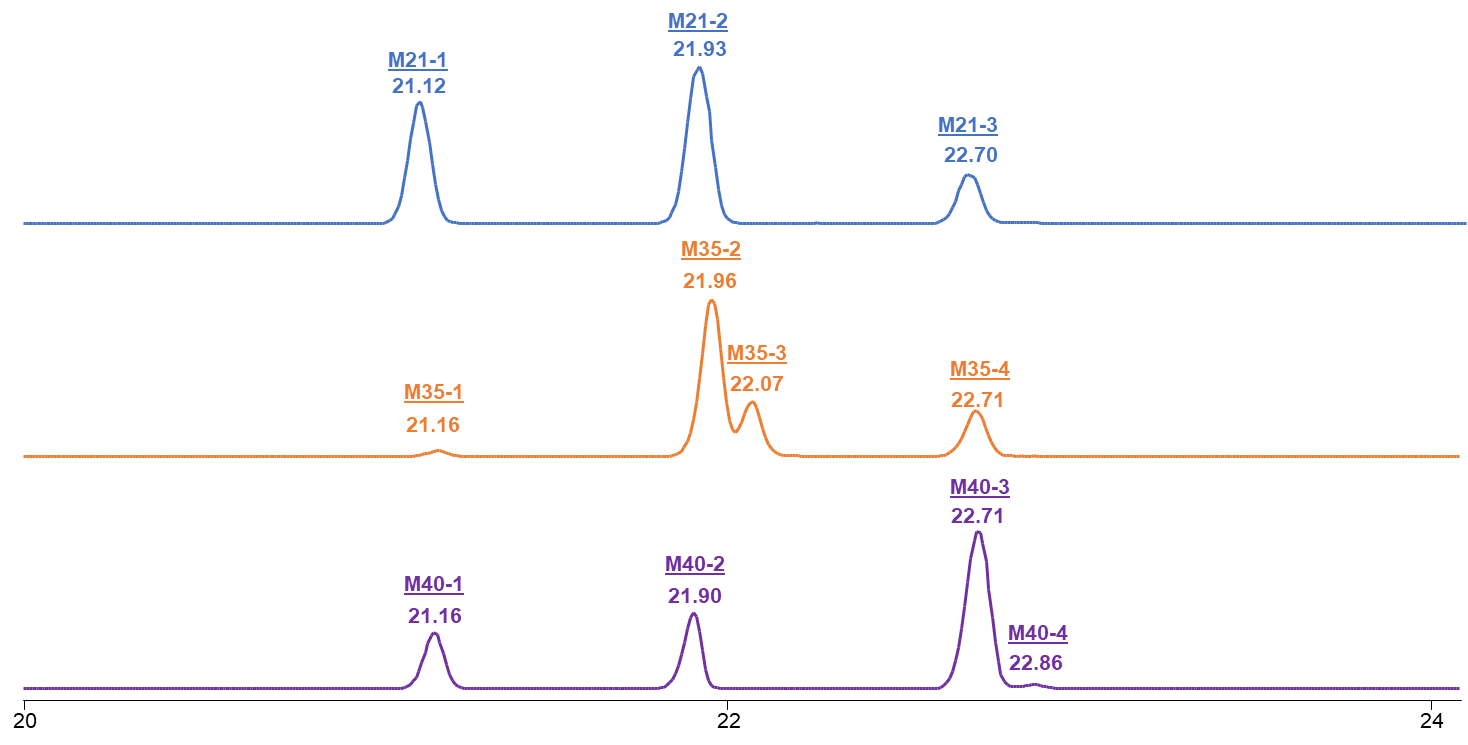


**Figure S8:** Extracted ion chromatograms of the m/z 1038.2 corresponding to the [M-H]- of the pentasacharide in sample M21 (blue) M35 (orange) and M40 (purple) between 20 and 24 min.

**
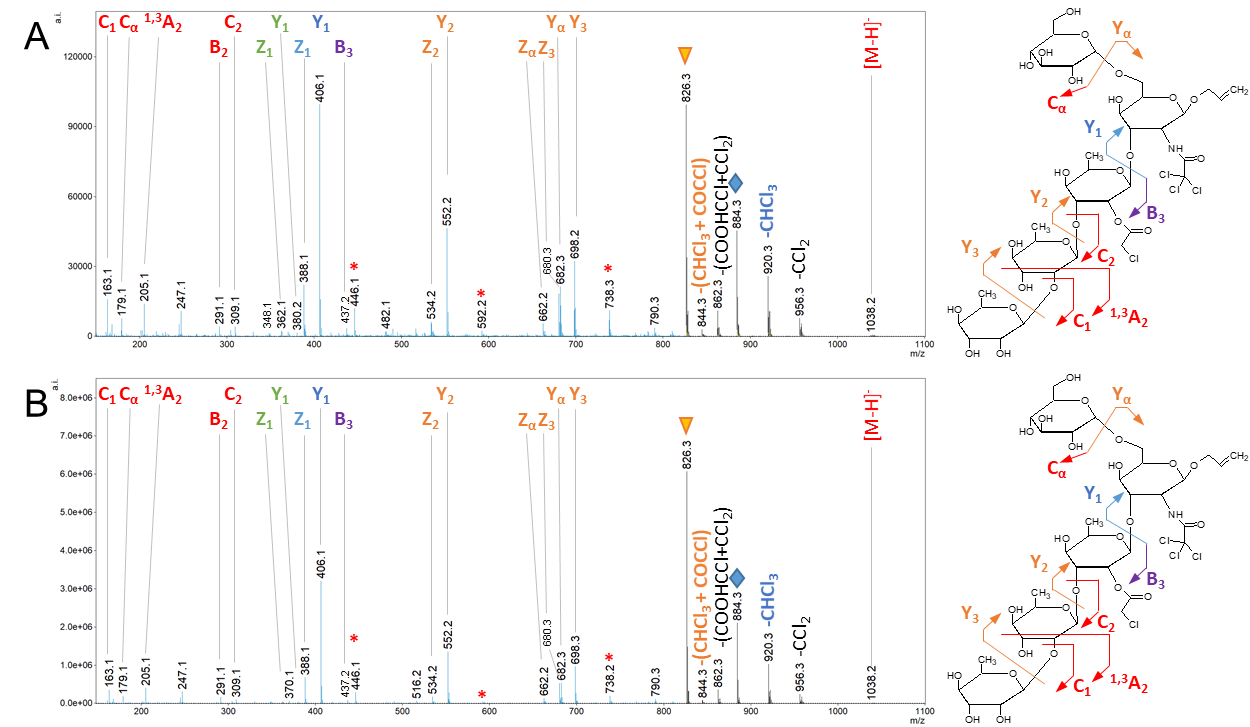
**

**Figure S9:** UHPLC-ESI-MS/MS validation of the structure of **P1** as **ABC’D’[E(1→6)]** isolated as [M-H]^-^ at m/z 1038.21 in samples M35 (A) and M40 (B) at *t_R_* = 22.71 min. The blue area of the spectrum is enlarged by a factor of 6 in the intensity axis. Annotations in red correspond to intact product ions. Annotation in green correspond to product ions with a loss of COCl_3_. Annotation in blue correspond to product ions with a loss of CHCl_3_. Annotations in purple correspond to product ions with a loss of COCCl. Annotation in orange correspond to product ions with a loss of CHCl_3_ + COCl_3_. All these labile function losses are in agreement with the structure. Red * indicate consecutive fragmentations, [▽](https://les-raccourcis-clavier.fr/faire-figure-triangle-clavier/#trianglebas) indicate H_2_O loss, ◊ indicate HCl loss. For clarity, only one fragment per pair (B, C and Y, Z) was reported on the annotated structures.


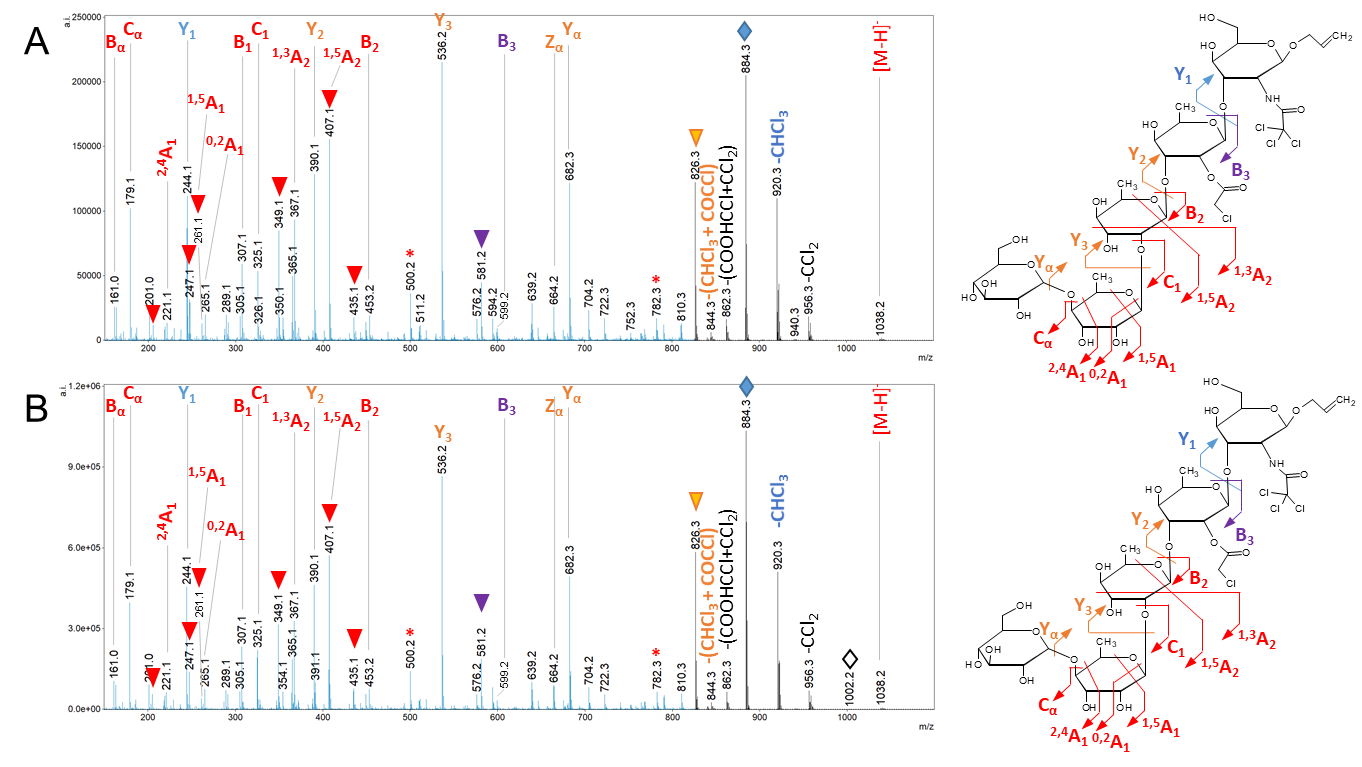


**Figure S10:** UHPLC-ESI-MS/MS validation of the structure of **P2** as **A[E(1→3)]BC’D’** isolated as [M-H]^-^ at m/z 1038.21 in samples M35 (A) at *t_R_* = 21.96 min and M40 (B) at *t_R_* = 21.90 min. The blue area of the spectrum is enlarged by a factor of 10 in the intensity axis. Annotations in red correspond to intact product ions. Annotation in blue correspond to product ions with a loss of CHCl_3_. Annotations in purple correspond to product ions with a loss of COCHCl. Annotation in orange correspond to product ions with a loss of CHCl_3_ + COHCl_3_. All these labile function losses are in agreement with the structure. Red * indicate consecutive fragmentations, [▽](https://les-raccourcis-clavier.fr/faire-figure-triangle-clavier/#trianglebas) indicate H_2_O loss, ◊ indicate HCl loss. For clarity, only one fragment per pair (B, C and Y, Z) was reported on the annotated structures.


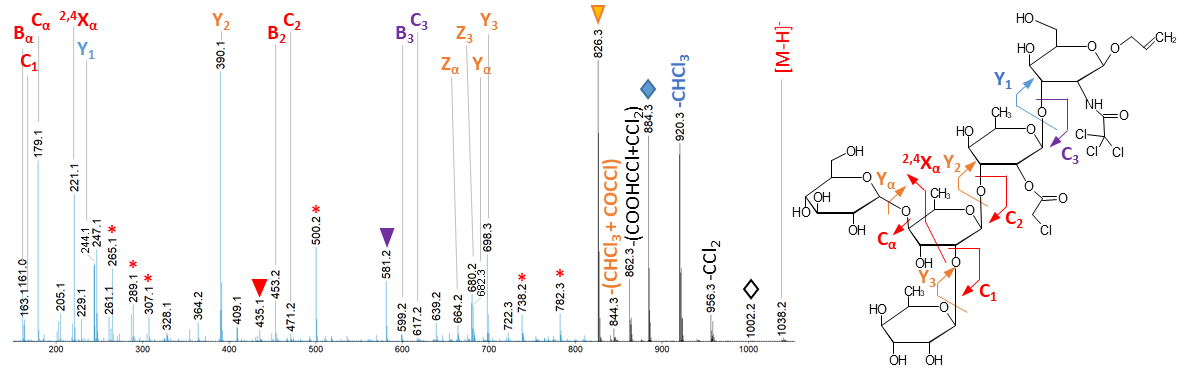


**Figure S11:** UHPLC-ESI-MS/MS spectrum of the pentasacharide isolated as [M-H]- at m/z 1038.21 at *t_R_* = 21.16 min in samples M40 validated as OH-4_B_. The blue area of the spectrum is enlarged by a factor of 4 in the intensity axis. Annotations in red correspond to intact product ions. Annotation in blue correspond to product ions with a loss of CHCl3. Annotations in purple correspond to product ions with a loss of COCHCl. Annotation in orange correspond to product ions with a loss of CHCl3 + COHCl3. All these labile function losses are in agreement with the structure. Red * indicate consecutive fragmentations, ▽ indicate H2O loss, ◊ indicate HCl loss. For clarity, only one fragment per pair (B, C and Y, Z) was reported on the annotated structure.

### **Supporting Tables**

**Table S1:** The table shows the Shannon entropy values for the redesignable positions, the conserved L627 and A672 are coloured in red color.

| Residue | H_(X)_ |
| --- | --- |
| G594 | **0.60** |
| W595 | **0.53** |
| F596 | **0.18** |
| L624 | **0.3** |
| E625 | **0.15** |
| F626 | **0.38** |
| L627 | **0** |
| L628 | **0.10** |
| A672 | **0** |

| I673 | **0.15** |
| --- | --- |
| S674 | **0.02** |
| F675 | **0.06** |
| A710 | **0.33** |
| P711 | **0.33** |
| R731 | **0.04** |
| D732 | **0.4** |
| K735 | **0.37** |
| K789 | **0.28** |
| D790 | **0.26** |

| I791 | **0.18** |
| --- | --- |
| Q792 | **0.09** |
| D793 | **0.36** |
| I796 | **0.39** |
| H797 | **0.55** |
| I798 | **0.10** |
| M810 | **0.32** |
| S811 | **0.57** |

**Table S2.** Summary of the mutations introduced in mutants of each group at the 27 re-designable residues. A blank indicates that the amino acid of the parental wild-type enzyme was conserved.


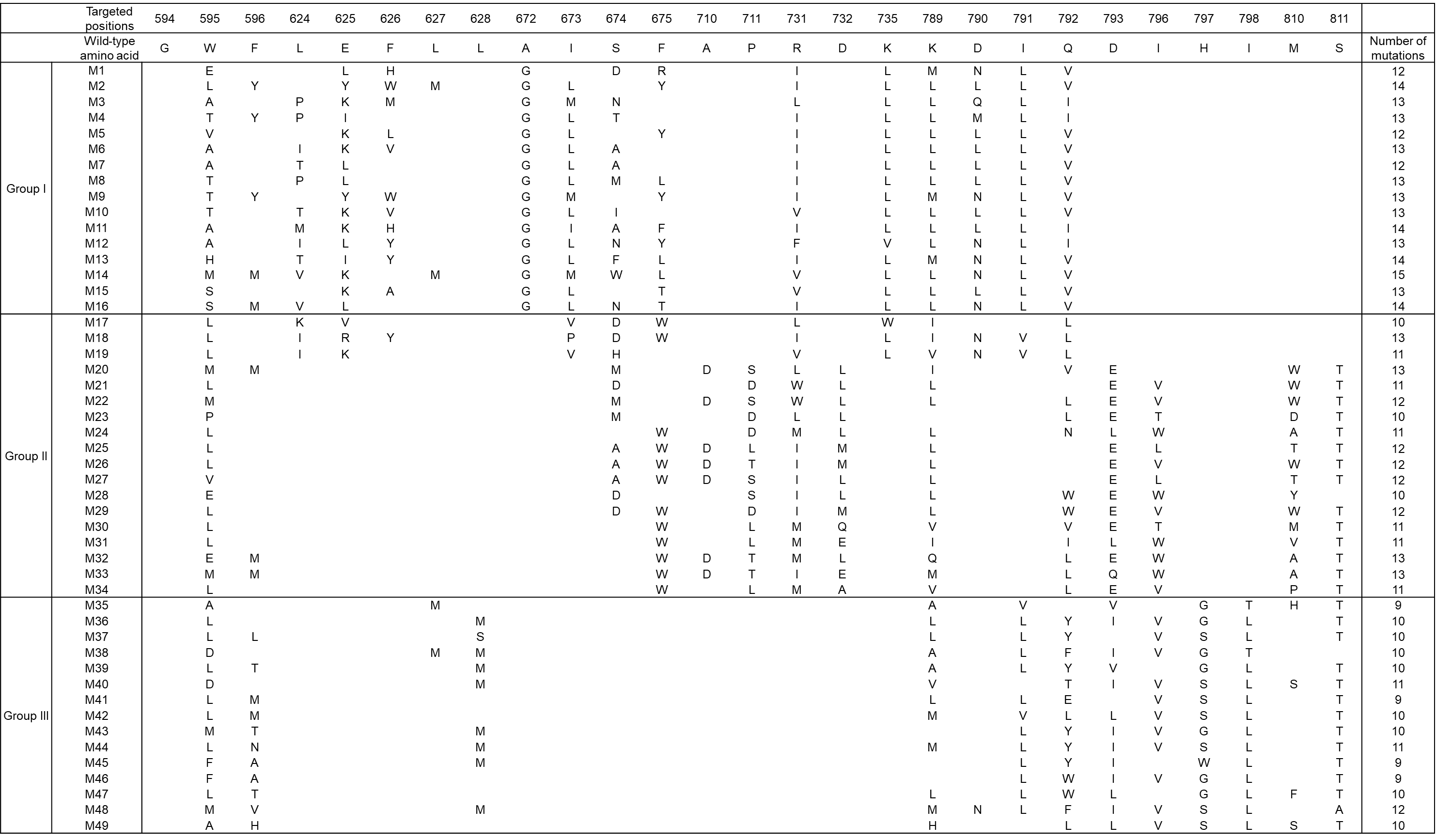


**Table S3.** The five plasmids encoding various sets of chaperone proteins tested in this study. Three chaperone sets co-expressed with BRS-B Δ2 mutants enabled soluble expression.

| Plasmid | Chaperone proteins encoded | Soluble expression detected |
| --- | --- | --- |
| pGRO7 | groES, groEL | No |
| pG-Tf2 | groES, groEL, tig | No |
| pTf16 | tig | Yes |
| pKJE7 | dnaK, dnaJ, grpE | Yes |
| pG-KJE8 | dnaK, dnaJ, grpE, groES, groEL | Yes |

**References**

1. Onufriev, A., Bashford, D. & Case, D. A. Exploring protein native states and large-scale conformational changes with a modified generalized born model. *Proteins* **55**, 383–94 (2004).

2. Weiser, J., Shenkin, P. S. & Still, W. C. Approximate atomic surfaces from linear combinations of pairwise overlaps (LCPO). *J. Comput. Chem.* **20**, 217–230 (1999).

3. Daudé, D., Topham, C. M., Remaud-Siméon, M. & André, I. Probing impact of active site residue mutations on stability and activity of Neisseria polysaccharea amylosucrase. *Protein Sci.* **22**, 1754–1765 (2013).

4. Gasteiger, E. *et al.* ExPASy: The proteomics server for in-depth protein knowledge and analysis. *Nucleic Acids Res.* **31**, 3784–3788 (2003).
